# Real-time dynamic monitoring and multiplex PCR identification of vector mosquitoes in Zhejiang, China

**DOI:** 10.1371/journal.pntd.0013129

**Published:** 2025-05-27

**Authors:** Qing He, Qinbao Lu, Ningzi Xie, Xiaohua Liu, Xinyi Wang, Jinren Pan, Sofia Litchev, Yuxin Hu, Xiaodong Li, Bin Zheng, Junfen Lin, Enfu Chen, Xiao-Guang Chen, Xiaohong Zhou, QingMing Kong, Shaohong Lu

**Affiliations:** 1 School of Basic Medicine and Forensics, Engineering Research Center of Novel Vaccine of Zhejiang Province, Zhejiang Provincial People’s Hospital (Affiliated People’s Hospital), Hangzhou Medical College, Hangzhou, China; 2 Zhejiang Provincial Center for Disease Control and Prevention, Hangzhou, China; 3 Institute of Tropical Medicine, Department of Pathogen Biology, School of Public Health, Southern Medical University, Guangdong Provincial Key Laboratory of Tropical Disease Research; Key Laboratory of Prevention and Control for Emerging Infectious Diseases of Guangdong Higher Institutes; Key Laboratory of Infectious Diseases Research in South China, Ministry of Education, Guangzhou, China; 4 Department of Chemistry & Biochemistry, University of California, Los Angeles, California, United States of America; 5 School of Laboratory Medicine and Bioengineering, Zhejiang Provincial People’s Hospital (Affiliated People’s Hospital), Hangzhou Medical College, Hangzhou, China; Egerton University, KENYA

## Abstract

The monitoring and identification of mosquito vectors are crucial for controlling the transmission of mosquito-borne diseases. Traditional mosquito morphological identification and surveillance methods, such as human landing catches, human-baited double net traps and BG-Sentinel mosquito traps, require a large amount of manpower but can only provide fragmented data. We utilized the MS-300, an internet-based vector mosquito monitor, to continuously capture and upload real-time data to cloud services across ten monitoring sites located in seven cities in Zhejiang Province, China from May to December 2023. A new multiplex PCR system was developed for amplifying the internal transcribed spacer 2 region, followed by employing both multiplex PCR and DNA barcoding techniques for detecting wild mosquitoes. A comprehensive monitoring of 9749 mosquitoes was conducted. The mosquito density gradually increased from May 2023, peaked around June 22^nd^, and then declined in a wave-like pattern. The mosquitoes have two peak activity times, the peak times may vary depending on different locations and seasons. The study showed the high specificity of a multiplex PCR system in distinguishing six mosquito species: *Aedes albopictus*, *Aedes aegypti*, *Culex pipiens pallens*, *Armigeres subalbatus*, *Anopheles sinensis* and *Anopheles anthropophagus*. Notably, the sensitivity of detecting *An. anthropophagus* reached an impressive 1fg/µL. With the exception of *Ae. aegypti* and *An. anthropophagus*, all four other mosquito species have been identified in Zhejiang Province, with *Cx. p. pallens* being the predominant population. The results were highly consistent with DNA barcoding technology. The MS-300 continuously and automatically monitors mosquito population density and activity, providing effective guidance for mosquito control based on the environment and reducing labor costs. Our newly established multiple PCR system enables precise identification of crucial vector mosquitoes, facilitating a comprehensive understanding of population structures across diverse regions for selecting effective control measures.

## Author summary

Surveillance of mosquito vectors has always been an indispensable component in the prevention and control of mosquito-borne viral and parasitic diseases. However, traditional mosquito surveillance methods like human landing catches, human-baited double net traps, BG-Sentinel mosquito traps are labor-intensive and provide limited data. In particular, the method of human landing catches is easily influenced by human and environmental factors, posing infection risks. We deployed an internet-based vector mosquito monitor called MS-300 to continuously track vector populations at ten locations in Zhejiang province over eight months. The data revealed variations in mosquito vector activity across different regions and seasons, likely influenced by population structure. The initial stages of mosquito vector identification primarily relied on morphological methods; however, inaccurate identification remains likely due to phenotypic plasticity and genetic variability. To enhance species identification, we have developed a multiplex PCR system targeting key vectors including *Aedes albopictus* and *Aedes aegypti*, which transmit Dengue virus or Zika virus, as well as *Anopheles sinensis* known as a malaria vector. This approach not only reduces the manpower requirements for mosquito vector control but also provides continuous and accurate data that offers crucial support for the prevention and management of mosquito-borne diseases.

## Background

In recent years, there has been a global upsurge in mosquito-borne diseases, necessitating the prioritization of transmission prevention and control as a critical public health concern. Studies conducted in North or South America have detected the presence of Dengue virus (DENV), Zika virus (ZIKV), and La Crosse virus in larvae and adults of *Aedes albopictus* (Skuse, 1897) [1–3]. The global distribution of ZIKV has expanded since 2015, particularly in the Americas. As of July 2019, microcephaly and other central nervous system malformations associated with ZIKV infection have been reported in 87 countries and territories [4]. Likewise, in China, the four anopheline species, namely *Anopheles sinensis* (Wiedemann, 1828), *Anopheles anthropophagus* (Baisas & Hu, 1936), *Anopheles minimus* (Theobald), and *Anopheles dirus* (Peyton & Harrison), are widely recognized as the primary vectors responsible for plasmodium transmission [5]. In particular, *An. sinensis* is the most widely distributed species in malarial outbreak areas of central China [6]. The *Culex pipiens pallens* (Harbach, 2012) complex mosquitoes also play crucial roles in the transmission of various pathogens, including the West Nile virus, St. Louis encephalitis virus, *Plasmodium spp.* responsible for avian malaria, and filarial worms responsible for elephantiasis [7]. *Armigeres subalbatus* (Coquillett, 1898) is also a potential vector for ZIKV [8].

In Zhejiang Province, important vector mosquitoes such as *Ae. albopictus*, *Ar. subalbatus, An*. *sinensis,* and *Cx. p. pipiens pallens* are present [9]. *Ae. aegypti* and *Ae. albopictus* mosquitoes are the main vectors for transmitting DENV [10,11]. Unlike *Ae. aegypti*, which cannot survive in cold winters, *Ae. albopictus* can endure temperate regions during its egg stage [11]. This factor may partially account for the limited outbreaks of dengue fever in temperate areas [12]. However, given the ongoing global climate change, it is crucial to monitor and address concerns regarding the potential of *Ae. aegypti* acquiring similar adaptive abilities as *Ae. albopictus* in sustaining populations within temperate regions. This is the reason why, despite few *Ae. aegypti* mosquitoes in temperate regions such as Zhejiang province, their inclusion in our monitoring scope remains imperative. They can transmit a range of diseases, posing a significant potential threat to human life and health. Therefore, it is crucial to monitor these vector mosquitoes in the long term, especially in epidemic areas.

**Fig 1:**
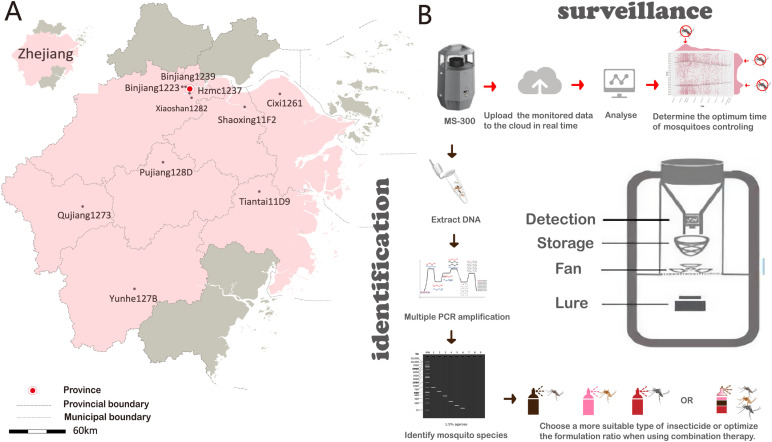
The placement of the MS-300 monitors, its internal structure, and the surveillance and identification flowchart. **(A)** The pink area on the map indicates the cities where monitoring points are located, while the gray area represents cities where no monitoring points have been established. The map data we used is derived from the public information of government departments (https://zhejiang.tianditu.gov.cn/standard). **(B)** Schematic diagram of the internal structure of the MS-300 monitor [26]. The operational mechanism is as follows: The independently developed insect lure volatilizes due to the fan-generated airflow at the bottom, attracting mosquitoes to the infrared detection window above. They are then drawn into the device through the detection window for automatic counting and fall into a storage net bag. The data is transmitted in real time to the cloud server via 4G. The icons for Culex pipiens mosquito, fever mosquito, house mosquito, tiger mosquito, and PCR cycle were provided by DBCLS (https://togotv.dbcls.jp/en/pics.html). The microtube-open-translucent icon was provided by Servier through https://smart.servier.com/. All these icons are derived from open-source resources available at https://bioicons.com.

Monitoring mosquito density is an essential process for making informed decisions regarding mosquito vector control. The existing methods for monitoring mosquito vectors include human landing catches (HLC) [13], human-baited double net traps (HDN), BG-Sentinel mosquito traps (BGS) [14], autocidal gravid ovitrap (AGO) [15], Gravid Aedes Trap (GAT) [16] and Mosquito Magnet (MM) [17]. The HLC method is widely recognized as the gold standard for adult mosquito surveillance. However, considering the potential health risks to collectors, particularly in areas affected by epidemics, it is not advisable to employ this method [18,19]. A further limitation of HLC is the variability in collecting skills among different collectors, which may also result in varying degrees of attractiveness to mosquitoes [20]. The primary role of vector surveillance is to ensure the control of science-based evidence, effectiveness of results, real-time data reporting, and subsequent adjustment of control strategies and methods based on evaluation reports. BGS traps have been used widely and are reported to be effective at estimating vector mosquito densities [21–24], but they still require manual counting, which requires a tremendous amount of manpower. Zhejiang Province has historically been a high-risk area for the outbreak of Japanese encephalitis [25], and continuous monitoring of mosquito vectors has consistently remained an imperative task. However, the data obtained through light trap surveillance exhibits intermittent patterns. The MS-300 monitor invented by Chen’s group [26] can monitor mosquito density around the clock and can automatically count and upload data in real-time to the cloud. The internal structure of the MS-300 monitor is illustrated in Fig 1B. The operational mechanism is as follows: The mosquito attractant Mix-5 was independently developed by Xie et al. [27], is a blend of human odors that exhibits exceptional efficacy in trapping vector mosquitoes and is specifically designed for luring and capturing these mosquitoes. The attractants volatilize at the bottom of the device due to the airflow generated by the fan, effectively guiding mosquitoes toward the infrared detection window located above. Subsequently, the mosquitoes are drawn into the device by the airflow and pass through the infrared detection window as they fall into the storage net bag for detection and automatic counting. The collected counting data are transmitted to the cloud server in real time via the 4G network, thereby achieving real-time monitoring of mosquito density. Furthermore, multiple sensors are incorporated within the device to continuously monitor micro-environmental fluctuations throughout the day. Not only does it save a lot of manpower, but also allows for continuous monitoring data, bringing new breakthroughs in technology to mosquito vector surveillance work.

Comprehending the composition of mosquito species is an indispensable and pivotal step towards proficiently managing mosquito vectors in order to ensure effective control measures. The early stages of mosquito vector identification mainly relied on morphological methods, but inaccurate identification can easily occur due to phenotypic plasticity and genetic variability [28]. However, due to the continuous operation of the fan in the MS-300 monitor, the samples collected through this device often lose their morphological characteristics, thereby affecting the accuracy of determining the species of mosquitoes based on morphological characteristics. In recent years, DNA barcoding technology has been extensively utilized in species classification [29,30]. This molecular technique has been successfully applied to mosquitoes in various countries [28,31–35]. The DNA barcoding technology offers more precise and reliable identification outcomes than morphological identification. However, it involves additional processing steps, higher costs, and a longer identification timeframe. While DNA barcoding exhibits high accuracy in identifying a broader diversity of mosquito species, its adoption in primary health institutions is limited by certain constraints. In contrast, multiplex PCR technology demonstrates significant advantages over DNA barcoding in terms of procedural simplicity, turnaround time, and cost-effectiveness. This is particularly evident when dealing with mixed samples, where multiplex PCR demonstrates significant superiority. Moreover, its identification accuracy is comparable to that of DNA barcoding technology [36]. The MS-300 monitor, as a continuous monitoring tool, can capture a large number of mosquito vector samples. Compared with DNA barcode technology, which requires identification and sequencing of each sample one by one, the multiplex PCR technology is more economical and efficient in dealing with mixed samples. At the same time, from the perspective of mosquito vector control, the world’s prevalent mosquito-borne diseases are mainly transmitted by several key mosquito species. For these important mosquito species, the development of multiplex PCR technology can facilitate rapid identification, thereby providing a robust scientific foundation for subsequent elimination and control strategies.

The objective of this study was to utilize MS-300 monitors for monitoring mosquito vectors in Zhejiang Province and evaluating real-time mosquito density. Additionally, the study aimed to develop a multiplex PCR system capable of identifying key disease vectors, providing an efficient alternative to complex and multi-step DNA sequencing methods.

## Materials and methods

### Arrangement of monitoring points

The MS-300 monitors were used to monitor and capture mosquitoes across ten monitoring sites located in seven prefecture-level cities of Zhejiang Province, from May to December 2023. All the MS-300 monitors were positioned in environments where mosquito vector activities were present, and they were maintained at a fixed location throughout the entire monitoring duration. The spatial arrangement of each monitoring site is depicted in Fig 1A and Table 1.

**Table 1:** Geographical coordinates of each monitoring machine.

	Number of machine	Location	Coordinate	Quantity of detected mosquitoes
1	Binjiang1223	HangZhou	30.27°N,120.13°E	1888
2	Binjiang1239	HangZhou	30.19°N,120.17°E	865
3	Xiaoshan1282	HangZhou	30.15°N,120.24°E	2250
4	Hzmc1237	HangZhou	30.20°N,120.22°E	772
5	Shaoxing11F2	ShaoXing	30.05°N,120.86°E	441
6	Cixi1261	NingBo	30.19° N,121.28°E	470
7	Pujiang128D	JinHua	29.46°N,119.90°E	316
8	Yunhe127B	LiShui	28.11°N,119.57°E	233
9	Qujiang1273	QuZhou	28.98°N,118.96°E	2118
10	Tiantai11D9	TaiZhou	29.14°N,121.04°E	396

### Analysis of monitoring data

All monitoring data are preprocessed in Excel, including outlier screening. Specifically: (1) A histogram of daily mosquito counts is generated, and values exceeding 300 are reviewed. If high values occur within 0–3 hours with 98%-100% humidity and all recorded sizes are 1*1, these are considered raindrop errors and deleted. (2) Data are cleaned based on the number of occlusion gratings from the MS-300 monitor. Mosquitoes passing through the infrared window cause occlusions; flapping may increase the range of this occlusion. Combining the characteristics of the grating spacing and the fact that the body length of most mosquito vectors is less than 1 cm. Selecting data within the range of 1*1–4*4 is valid mosquito signal; other data is deleted as non-mosquito insect data. Finally, R version 4.4.2 is used for analysis and visualization. First, we will generate a scatter plot with date on the x-axis and time on the y-axis to analyze the yearly temporal distribution of mosquito occurrences. Next, we will use facet analysis to evaluate significant variations in mosquito activity patterns across locations. Finally, we will create time density maps for mosquito activities in different months at the same location to determine if peak periods shift with changing months.

### Source of mosquitoes

The five mosquito species utilized for the establishment of the multiplex PCR method in this study, namely *An. sinensis*, *An. anthropophagus*, *Ar. subalbatus, Ae. albopictus*, and *Ae. aegypti*, were sourced from strains preserved at the Tropical Disease Laboratory of the Department of Pathogen and Microbiology, School of Public Health, Southern Medical University. *Cx. p. pallens* was collected using MS-300 monitors [26] in Xihu District, Hangzhou City, Zhejiang Province. The mosquito samples used for constructing the multiplex PCR system have all been pre-identified by DNA barcoding technology for species.

### DNA extraction

The collected mosquito samples were anesthetized at -20°C for 10 minutes before undergoing morphological classification using the taxonomic key by Lu et al [37] under a stereomicroscope. Each individual mosquito was transferred to a 1.5ml EP tube for subsequent DNA extraction using the Insect DNA Kit (OMEGABio-Tek, D0926-01, Guangzhou, China) in accordance with to the manufacturer’s standard protocol. To ensure no cross-contamination occurs, only one type of mosquito’s DNA was extracted per batch, and the resulting products were stored at -20°C.

### Primers

The internal transcribed spacer 2 (ITS2) sequences from various mosquito species were obtained from the NCBI Genbank database. Homology alignment was then performed using SnapGene software version 4.36, with exclusion of regions homologous to primer binding sites. One end of each primer was located in a non-homologous region to amplify a single band for each mosquito species, and product sizes were separated by more than 50 bp to facilitate differentiation during agarose gel electrophoresis. Each primer should not have any interconnections or self-connections, and their annealing temperatures should be as close as possible. Whenever possible, common primers were used to minimize their number and ensure stability in the multiplex PCR system. Using MFEprimer3 software, nine primers were designed to accurately identify *An. sinensis*, *An. anthropophagus*, *Cx. p. pallens*, *Ae. albopictus*, *Ar. subalbatus,* and *Ae. aegypti* without detecting self-dimerization or cross-reactivity among the primers. All the primers used in this study were synthesized by Shanghai Sangon Biotech Co., Ltd. The details of the primers, including their respective amplification lengths, are shown in Table 2.

**Table 2:** Primers used in multiplex PCR and DNA barcoding.

Name of Primers^*^	Primer sequences (5`-3`)	Tm (°C)	LOCUS	PCR Product size (bp)	Socrce
*An. sinensis* F	TACGCAAATAATCATTGTATGGAACCC	54.8	GU384693	684	This study
*An. sinensis* R	TAGGGTCAAGGCATACAGAAGG	56.3			
*Cx. p. pallens* F	TGTTTCTTCTTTGAACTCGCTCGC	57.8	U22116	527	This study
*Cx. p. pallens* R	TTATCGCTTGTCGTTCGCTGC	58.5			
*An. anthropophagus* F	AACTACGCAGTGATTGGTGC	55.7	AF543860	379	This study
*An. anthropophagus* R	TTCTCAGGTAGAACTTGCATCTTCC	56.3			
*Ae. albopictus* F	AGTGGCAGTGAGCTTAGTAC	54.1	L22060	251	This study
*Ae. albopictus* R	TAGTCACACATTATTTGAGGCCTAC	53.9			
*Ar. subalbatus* F	ACCGCGGTTGATGAATACAT	54.1	KU497621	156	This study
*Ar. subalbatus* R	TAGTCACACATTATTTGAGGCCTAC	53.9			
*Ae. aegypti* F	ACCGCGGTTGATGAATACAT	54.1	GU980956	108	This study
*Ae. aegypti* R	TAGTCACACATTATTTGAGGCCTAC	53.9			
LCO1490	GGTCAACAAATCATAAAGATATTGG	49.2	–	710	[38]
HCO2198	TAAACTTCAGGGTGACCAAAAAATCA	54.2			

* F represents the forward primer, and R represents the reverse primer.

### Establishment and optimization of multiplex PCR conditions

The components of total multiplex PCR reaction are as follows: Flash HS PCR Master Mix (code No.AG12301, Accurate Biotech, China) at a concentration of 2x (12.5 μL), primers at a concentration of 10μM (0.5μL each), DNA template (2μL), and RNase-free water adjusted to reach a total volume of 25 μL. The amplification process begins with an initial denaturation step at 98°C for 2 min, followed by 35 cycles. Each cycle includes a denaturation step at 98°C for 10s, an annealing step at 59.3°C ~ 64.1°C for 20s, and an extension step at 72°C for 10s. Finally, there is a further extension stage where the temperature is maintained constant at 72°C for 5 min. The optimal annealing temperature for multiplex PCR reactions was evaluated at eight different temperature points: 59.3°C to 64.1°C (59.3°C, 59.8°C, 60.5°C, 61.3°C, 62.1°C, 62.8°C, 63.5°C and 64.1°C). Gel electrophoresis was conducted utilizing 1.5% agarose gel in combination with 0.5 × TBE buffer 100V voltage over a period of 45–50 min.

### Experiments on sensitivity and specificity of multiplex PCR

The DNA templates of each mosquito species were diluted using a double steam water gradient, resulting in ten concentrations: 10ng/µL, 1ng/µL, 100pg/µL, 10pg/µL, 1pg/µL, 100fg/µL, 10fg/µL, 1fg/µL, 0.1fg/µL and 0.01fg/µL. After amplification using the optimized multiplex PCR reaction system, each primer pair was assessed for sensitivity based on the observed band intensity during agarose gel electrophoresis.

Two methods were used to assess the specific binding and amplification of DNA templates in the multiplex PCR system, we utilized two approaches to assess the amplification products: verifying their size via gel electrophoresis and subjecting them to unidirectional sequencing at Shanghai Sangon Biotech Company followed by blast analysis on NCBI database.

### Detection of field samples

To assess the accuracy and feasibility of multiplex PCR, the same batch of field mosquito samples were identified using both DNA barcoding technology and multiplex PCR technology simultaneously. Specifically, we collected mosquitoes captured by the MS-300 monitor at each monitoring point from May to December, with the exception of the Binjiang1239 and Tiantai11D9 monitoring points. All the initially identified mosquito vectors were systematically classified into four distinct categories: *Aedes spp.*, *Culex spp.*, *Anopheles spp.*, and *Armigeres spp.*, based on their morphological characteristics, including body color and wing patterns. Then stratified random sampling was carried out within these four categories. Ultimately, we collected a total of 140 field samples, among which 38 *Aedes spp*., 85 *Culex spp.*, 15 *Armigeres spp.*, and 2 *Anopheles spp.* were selected and identified using two methods. In DNA barcoding technology, after Cytochrome coxidase subunit 1 (*cox1*) gene amplification and sequencing, the identification results are obtained by comparing the sequencing results with the NCBI database. The primer sequences utilized for *cox1* gene amplification were showed in Table 2. The total volume of the PCR reaction was 25μL [28], comprising Flash HS PCR Master Mix (code No.AG12301, Accurate Biotech, China) at a concentration of 2× (12.5μL), primers at a concentration of 10μM (0.5μL each), DNA template (2μL), and RNase-free water adjusted to reach the final volume. The amplification process included an initial denaturation at 98°C for 2 min, 35 cycles (denaturation at 98°C for 10s, annealing at 46.7°C for 20s, and extension at 72°C for 10s), and an extension stage where the temperature was maintained constant at 72°C for five minutes. The amplified fragments were analyzed using agarose gel electrophoresis with ethidium bromide staining under UV light to assess their integrity. PCR products were sent to Shanghai Sangon Biotech for unidirectional sequencing and blast analysis was performed on NCBI. Perform sequence alignment and evolutionary analysis in MEGA (version 10.2.6) to compare the sequenced *cox1* gene sequences with those obtained from database (NCBI Genbank; www.ncbi.nlm.nih.gov/genbank) for other mosquito species, and generate a phylogenetic tree using the Neighbor-Joining method.

## Results

### Analysis of the monitoring data from MS-300 monitors

**Fig 2:**
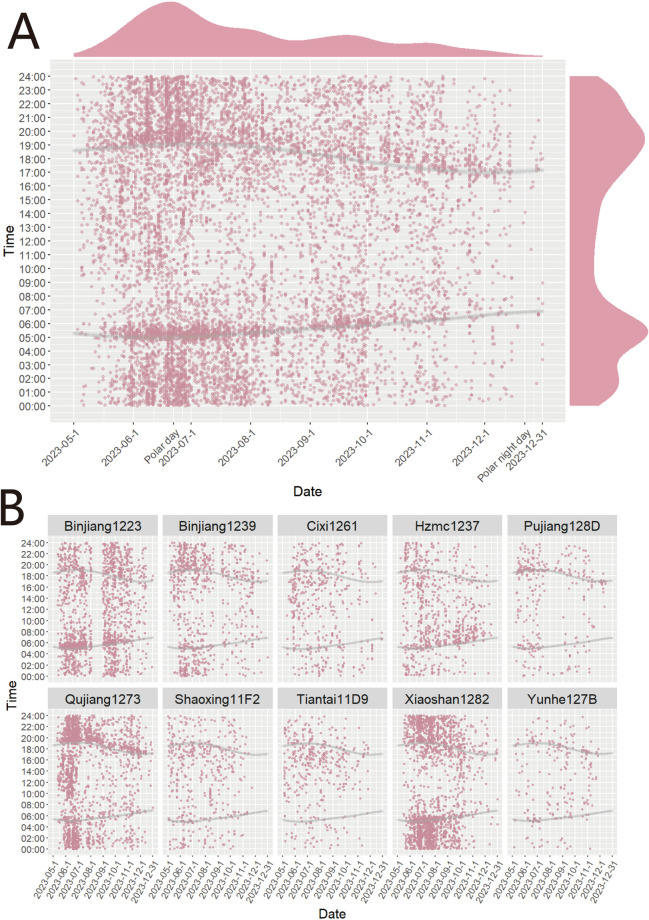
The scatter plot of mosquitoes monitored by all the MS-300 monitors from May 1^st^ to December 31^st^. Pink dots indicate mosquito appearance times, while gray dots represent sunrise and sunset times for each monitoring point (based on Beijing time). **(A)** Scatter plot displays the time and date of all mosquitoes detected at all the monitors. **(B)** Scatter plot displays the time and date of all mosquitoes monitored at each monitoring point. There were some data missing for Binjiang1233 from August 4^th^ to August 27^th^.

**Fig 3:**
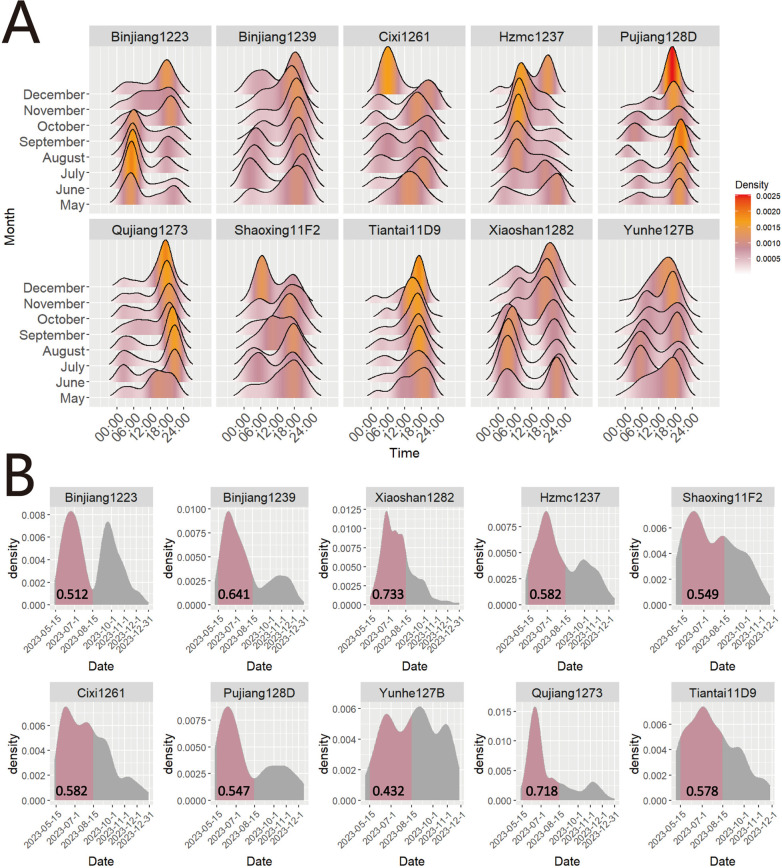
The density curves of mosquitoes monitored by all the MS-300 monitors from May 1^st^ to December 31^st^. (A) The density distribution of each monitor is showed throughout the monitoring period, on a monthly basis, for 24 hours per day. **(B)** The distribution of mosquito density from each monitor throughout the entire monitoring period. The pink area represents the area under the curve of mosquito vector density from May 15^th^ to August 15^th^, with the displayed numbers reflecting its proportion throughout the entire monitoring period. The calculation method is as follows: Divide the total number of mosquitoes recorded at each monitoring site from May 15^th^ to August 15^th^ by the total number of mosquitoes recorded throughout the monitoring period (from May 1^st^ to December 31^st^). There was a part of data missing for Binjiang1233 from August 4^th^ to August 27^th^, which resulted in an underestimation of the calculated ratio for Binjiang1233 as shown in Fig 3B.

Between May and December 2023, an extensive capture of 9749 mosquitoes was conducted utilizing MS-300 monitors in seven prefecture-level cities throughout Zhejiang Province. The detailed information of each recorded mosquito can be found in S1 File. The mosquito density gradually increased from May 2023, peaked around June 22^nd^, and then declined in a wave-like pattern. During the 24-hour monitoring period, we observed two distinct peaks of mosquito density: one from 04: 30–06: 30 in the morning and another from 18: 00–21: 00 in the evening (Fig 2A). We also analyzed the daily sunrise and sunset times to verify if the peak periods coincided with these time intervals. The results indicated that, regardless of whether it was June, when the mosquito vector density was highest, or November, when the density was much lower, the mosquito vector density within one hour before and after sunrise and sunset was significantly higher compared to other periods. We further analyzed the distribution of mosquito vectors in each location throughout the monitoring period (Fig 2B). Interestingly, we observed two peak periods of mosquito density within a day during the 24-hour monitoring. However, further analysis revealed non-uniform distribution of mosquito numbers during these periods. The research results indicate that the density of mosquito vectors exhibits significant variation across different locations, and their diurnal activity patterns also exhibit distinct characteristics. For instance, mosquito vectors at the locations of Binjiang1233 and Hzmc1237 monitors exhibit higher activity during the sunrise period; conversely, those at the locations of Binjiang1239, Qujiang1273, Shaoxing11F2, Tiantai11D9, and Cixi261 monitors show increased activity during the sunset period. Additionally, the monitoring data from Xiaoshan1282 indicate that mosquito vectors in this area are relatively active during the sunrise, sunset, and night periods. To investigate whether the active period of mosquito vectors varies seasonally, we categorized the mosquito vector data from each location by month and plotted all-day density curves to examine the trends in peak activity periods (Fig 3A). It has been revealed that the density of mosquito vectors during these peak periods fluctuates across different months at the same monitoring site. According to the data obtained from the Binjiang1233 monitoring site, the density of mosquito vectors exhibited a predominant concentration before 6:00 during the period from May to September; whereas, from October to December, it was primarily concentrated after 18:00. Data from the Qujiang monitoring site showed a consistent pattern where the peak density of mosquito vectors consistently occurred after 18:00 between June and December. However, it is noteworthy that in June, the peak appeared at a later time compared to subsequent months when it gradually advanced earlier. Each monitoring point shows distinct variations in mosquito vector density throughout the monitoring period. However, except for the data fromYunhe127B monitor, all other monitors recorded mosquito vector counts exceeding half of the total number during the entire monitoring period from May 15^th^ to August 15^th^. Notably, Xiaoshan1282 and Qujiang1273 monitor reached percentages of 73.3% and 71.8%, respectively (Fig 3B).

### Optimized multiplex PCR conditions

The results of individual PCR tests for the DNA of each reference mosquito revealed successful amplification of specific gene products. These products were 684 bp for *An. sinensis*, 522 bp for *Cx. p. pallens*, 379 bp for *An. anthropophagus*, 251 bp for *Ae. albopictus*, 156 bp for *Ar. subalbatus* and 108 bp for *Ae. aegypti*. When the annealing temperature of the multiplex PCR was 62°C, the amplified bands of six target genes of *An. sinensis*, *An. anthropophagus*, *Cx. p. pallens*, *Ae. albopictus*, *Ar. subalbatus* and *Ae. aegypti* were uniform, concentrated and highly specific. The subsequent tests were carried out at 62°C.

### Multiplex PCR sensitivity and specificity experiments

**Fig 4:**
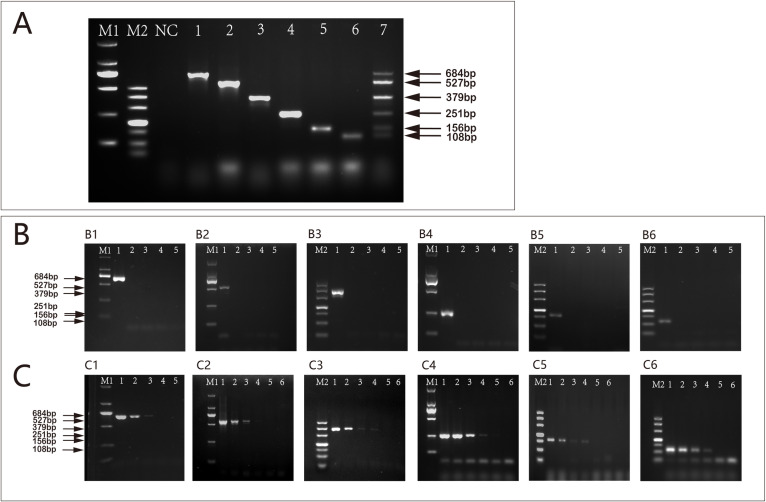
The sensitivity and specificity of multiplex PCR. LaneNC: RNase free water; Lane M1, GL2000 DNA marker; Lane M2, DL500 DNA marker; **A: Results of multiplex PCR reactions of six mosquito species.** Lane1 ~ 7, *An. sinensis*; *Cx. p. pallens*; *An. anthropophagus*; *Ae. albopictus*; *Ar. subalbatus*; *Ae. aegypti*; mixed DNA templates. **B: Results of Multiplex PCR Specificity Experiment. B1 (*An. sinensis* Primers)**: Lane1, *An. sinensis*; Lane2 ~ 5, *Cx. p. pallens*; *An. anthropophagus*; *Ae. albopictus*; *Ae. aegypti*; **B2 (*Cx. p. pallens* Primers)**: Lane1, *Cx. p. pallens*; Lane2 ~ 5, *An. sinensis*; *An. anthropophagus*; *Ae. albopictus*; *Ae. aegypti*; **B3 (*An. anthropophagus* Primers)**: Lane1, *An. anthropophagus*; Lane2 ~ 5, *An. sinensis*; *Cx. p. pallens*; *Ae. albopictus*; *Ae. aegypti*; **B4 (*Ae. albopictus* Primers)**: Lane1, *Ae. albopictus*; Lane2 ~ 5, *An. sinensis*; *Cx. p. pallens*; *An. anthropophagus*; *Ae. aegypti*; **B5 (*Ar. subalbatus*)**: Lane1 ~ 5, *Ar. subalbatus* Primers; *An. sinensis* Primers; *Cx. p. pallens* Primers; *An. anthropophagus* Primers; *Ae. albopictus* Primers; **B6 (*Ae. aegypti* Primers)**: Lane1, *Ae. aegypti*; Lane2 ~ 5, *An. sinensis*; *Cx. p. pallens*; *An. anthropophagus*; *Ae. albopictus*; **C: Results of Multiplex PCR Sensitivity Experiment. C1** (***An. sinensis***), **C2 (*Cx. p. pallens*)**, **C3 (*An. anthropophagus*)**: Lane1 ~ 6: 1pg/µL; 100fg/µL; 10fg/µL; 1fg/µL; 0.1fg/µL; 0.01fg/µL; **C4 (*Ae. albopictus*)**: Lane1 ~ 6: 10pg/µL; 1pg/µL; 100fg/µL; 10fg/µL; 1fg/µL; 0.1fg/µL; **C5 (*Ar. subalbatus*)**, **C6 (*Ae. aegypti*)**: Lane1 ~ 6: 100pg/µL; 10pg/µL; 1pg/µL; 100fg/µL; 10fg/µL, 1fg/µL.

Sensitivity test results of the reference mosquito DNA demonstrated that, multiplex PCR assay was capable of properly identifying the presence of DNA at the following lowest concentration, 10fg/µL for *An. sinensis* (Fig 4C1), 10fg/µL for *Cx. p. pallens* (Fig 4C2), 1fg/µL for *An. anthropophagus* (Fig 4C3), 10fg/µL for *Ae. albopictus* (Fig 4C4),100fg/µL for *Ar. subalbatus* (Fig 4C5), 100fg/µL for *Ae. aegypti* (Fig 4C6). Each template exhibited specific binding to its corresponding primer and successfully amplified the desired band of expected size, consistent with the amplification product from a single primer (Fig 4B1–4B6). The specificity of this multiple PCR method also has been validated through both electrophoresis and sequencing analyses. The detailed sequencing results can be found in S2 File.

### Detection and identification results of field samples

**Fig 5:**
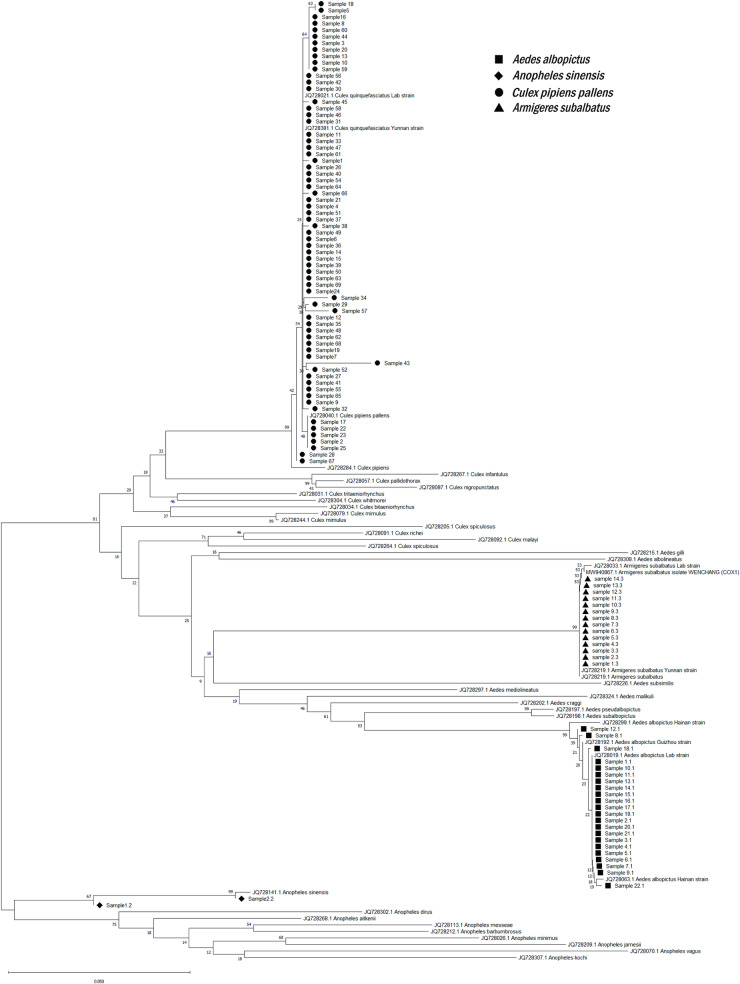
The phylogenetic tree of field mosquitoes.

During the identification process, out of 140 samples, 34 could not be identified by DNA barcoding technology due to suboptimal quality. The remaining 106 samples were successfully identified using multiplex PCR and DNA barcoding techniques. It was determined that 68 (64.15%) were *Cx. p. pallens*, 22 (20.75%) were *Ae. albopictus*, 14 (13.21%) were *Ar. subalbatus*, and there were also 2 (1.89%) of *An. sinensis*. Selected amplification results are presented in Fig 4A. The phylogenetic tree of the *cox1* gene in positive samples is shown in Fig 5. The detailed sequencing results can be found in S3 File.

## Discussion

The monitoring of mosquito vectors aims to quantify population size and species composition, establish spatiotemporal abundance patterns, track diseases transmission and provide data-driven guidance for effective mosquito control strategies. In this study, real-time dynamic monitoring data revealed variations in mosquito activity times across different locations in Zhejiang Province, despite being within the shared subtropical monsoon climate. This implies that when formulating targeted adjustments to mosquito control plans, it is crucial to consider specific geographical and environmental factors. The grating spacing of the detection window of the MS-300 monitor is 2 mm. and its detecting ranges from 2mm to 10mm. The body lengths of target mosquitoes such as *Aedes*, *Culex* and *Anopheles* mosquitoes are ranged from 3.5mm to 6.5mm. When the target mosquito goes through the detection window, its body would block the grating formed by infrared ray, and the blocking range from 1 to 4 would be recognized as the target mosquito. Because the grating of the MS-300 monitor is consisted of two directions, X-direction and Y-direction, the blocking range of 1*1–4*4 would be recognized as mosquitoes. However, this detection cannot distinguish non-target insects with similar body lengths to target mosquitoes. The MS-300 monitor had been developed as a trap special to vector mosquitoes by combined using an attractant, Mix-5. Mix-5 was developed by a mixture of human odors and displayed good effect to trap vector mosquitoes [27]. However, the field test for the MS-300 monitor showed that the non-target insects can still be trapped [26]. A total of 467 nontarget arthropods accounting for 11.1% of the total insects were caught in the garden and 28.1% in the residence respectively by the MS-300 monitor [26]. These results demonstrated that MS-300 monitor exhibits consistently sensitivity to mosquitoes across various environmental conditions and has a relatve high consistent rate bewtten the number of mosquitoes caught in the bag of MS-300 monitor and the data documented in the cloud, which suggest that MS-300 monitor can be used to monitor the seasonal dynamics of vector mosquitoes in the fields.

The data collected by MS-300 monitors in Zhejiang Province from May 2023 demonstrates a gradual increase in mosquito density, reaching its peak around June 22^nd^ before exhibiting a wave-like decline. In the Fig 3B, the proportion of mosquito vectors observed at each monitoring point during the period from May 15^th^ to August 15^th^ varies between 43.2% and 73.3% of the entire monitoring cycle. These findings suggest that targeted interventions for mosquito control should be implemented prior to the peak period on June 22^nd^, highlighting the necessity for intensified efforts in vector control between May 15^th^ and August 15^th^. A primary strategy for arbovirus outbreak control, such as dengue, is the use of synthetic chemicals with quick-action killing of adult vectors using space spraying [39]. The timing of spraying plays a crucial role in the effectiveness of spatial spraying, as we need to use insecticides when mosquitoes are active and exposed [40].

Different mosquito species exhibit distinct activity patterns across various times of day [41,42], and environmental factors also play a critical role in shaping these patterns [43]. At present, research findings indicate that the *Ae. albopictus* exhibits two distinct biting cycles. The initial biting cycle typically occurs between 05:00 and 08:00, while the subsequent biting cycle generally takes place from 16:00–19:00 [44,45]. However, in some studies, the biting activity of *Culex* mosquitoes such as *Culex Modestus* (Ficalbi, 1890) ranged from -0.5 to +2 hours after sunset, with peak biting occurring at +1 hour or exclusively feeding at night with peak activity between 22:00 and 00:00 [46,47]. Through our analysis and research, we have observed that the daily activity patterns of mosquito vectors display temporal and spatial variation depending on location and season. As shown in Fig 3A, mosquito activity at the Xiaoshan site displays two distinct peaks during morning and evening hours from May to September. However, after September, this activity shifts to a single peak in the evening. At the Qujiang monitoring site, while only an evening peak is observed from June to December, its occurrence time progressively shifts earlier with each passing month. According to the distinct activity patterns exhibited by different mosquito species, it is reasonable to speculate that these variations may stem from their unique composition of mosquito species. Furthermore, seasonal variations may prompt adaptive adjustments in the composition of mosquito species or their activity patterns. The aforementioned observations underscore the significance of tailoring disinfection efforts to suit specific local conditions and time periods, while ensuring regular updates and adjustments in accordance with changing environmental factors.

Our research also highlights the influence of photoperiod on mosquito behavior. From May to December 2023, there was a noticeable increase in mosquito activity occurring one hour after sunrise and one hour before sunset. Fig 2A illustrates a gradual reduction in daylight duration during this period, accompanied by a decreasing interval between morning and evening activity peaks. This photoperiod sensitivity a well-documented trait among many insect species, including mosquitoes, and its regulation involves maternally inherited genetic mechanisms [48,49]. This refined understanding of mosquito behavior, driven by both spatial and temporal variables, is essential for optimizing vector control strategies, ensuring effective and timely disinfection measures based on localized environmental conditions.

The MS-300 monitor offers real-time and long-term monitoring capabilities, allowing it to detect subtle continuous changes effectively. This makes it a valuable tool for guiding efforts mosquito vectors control efforts. Unlike traditional fixed-schedule insecticide application adjusting pest management to account for geographical, environmental, and seasonal variations significantly improves efficiency gains while simultaneously reducing labor demands and enhancing ecological sustainability. Crucially, this approach also minimizes reliance on human-based attraction methods, and freeing up substantial resources for other critical tasks.

The determination of the optimal disinfection time is just one crucial component in achieving effective mosquito control, which necessitates careful consideration of meteorological conditions, area selection, and choice of insecticides [50,51]. Further identification of mosquito vector species is essential for selecting appropriate insecticides, enabling more precise formulation and application. To achieve this, we have developed and refined multiple PCR techniques, ultimately achieving significant success in the ITS2 region, which is a phylogenetic marker for resolving taxonomic issues in mosquito species such as the Hyrcanus group due to its low intraspecific and high interspecific variability [52–57].

Our PCR primers, designed based on the ITS2 region, demonstrate high specificity when compared to the sensitivity of other four multiplex PCR assays at a concentration of 1pg/μL [58]. Additionally, the six-round PCR system we developed exhibits robust and remarkable stability even at a lower concentration of 1fg/µL. This system enables both single-sample and multiple-sample detection. Compared with DNA barcoding technology, multiplex PCR is more suitable for the rapid identification of large-scale samples due to its higher efficiency. Using this approach, we identified four mosquito species in Zhejiang Province, namely *Ae. albopictus*, *Cx. p. pallens*, *Ar. subalbatus* and *An. sinensis*; among them, *Cx. p. pallens* exhibited population dominance. To ensure the accuracy of our results, we further utilized DNA barcoding, which provided highly consistent findings with multiplex PCR. Multiplex PCR ensures accurate discrimination among species through the design of highly specific primers. However, mutations in the ITS2 region of wild mosquito populations may compromise primer recognition, thereby reducing the accuracy of multiplex PCR. This presents a common challenge when transitioning multiplex PCR technology from laboratory settings to field applications. In this study, we employed multiplex PCR to identify wild mosquitoes in Zhejiang Province. The results demonstrated that no recognition issues attributable to ITS2 mutations were encountered, indicating that this technology exhibits high reliability and practical application value. Although DNA barcoding can identify a greater diversity of species compared to multiplex PCR, it requires specific concentration thresholds for successful sequencing and cannot effectively process mixed samples—limitations that multiplex PCR does not possess. Despite the limited sample size in our random sampling, the identification results are consistent with those reported in previous studies [59]. However, if we want to obtain accurate and comprehensive information on mosquito vector composition, it is imperative to deploy additional monitors as mosquito species composition is significantly influenced by environmental factors. Therefore, a kind of mosquito species composition ratios would not adequately represent each location.

Our six-round PCR system not only matches the accuracy of DNA barcoding but also presents a more streamlined, cost-effective, and operationally efficient solution for mosquito control workers. When combined with the real-time monitoring capabilities of the MS-300 monitor, these PCR techniques overcome the limitations associated with traditional identification methods, providing a comprehensive and scientifically robust framework for managing mosquito vectors. This integration significantly enhances the precision and efficiency of vector control strategies, reducing labor while ensuring ecological sustainability.

## Conclusions

The MS-300 monitor offers continuous and automated monitoring of mosquito population density and activity, providing valuable guidance for mosquito control while significantly reducing labor costs. Through the utilization of a six-round PCR method developed in this study, we successfully identified four mosquito species in Zhejiang Province: *Ae. albopictus*, *Cx. p. pallens*, *Ar. subalbatus*, and *An. sinensis*; with *Cx. p. pallens* being the predominant species observed. This precise identification is crucial for assessing risks associated with mosquito-borne diseases and tailoring effective control strategies.

## Supporting information

S1 File All the data collected by each MS-300 monitor. (XLSX)

S2 File Sequencing results of the multiplex PCR products. (DOCX)

S3 File The sequencing results of the *cox1* gene of 106 field mosquito samples. (DOCX)
