# Supplementary material for: Real-time dynamic monitoring and multiplex PCR identification of vector mosquitoes in Zhejiang, China
Source: PLoS Negl Trop Dis. 2025 May 27;19(5):e0013129. doi: 10.1371/journal.pntd.0013129 (PMC12140415; doi:10.1371/journal.pntd.0013129)
Supplement: S2 File — (DOCX) [file pntd.0013129.s002.docx]

**Sequencing results of the multiplex PCR products**

| **species** | **sequencing results of the multiplex PCR products5`~3`** |
| --- | --- |
| *Anopheles sinensis* | ACTTATGGCACTAGAGAACACTACCCAGATTTGTTATGTTAGCGGGCTGGACAACAATAATACAGCAAACAAAGGTCAAACAATTATCACTCCAAGAGTGAGGCCACTCGGTGGTCAGATAAGCCTCAAGTTATGTGTGACAACCCCCTGAATTTAAGCATATTAATAAGGGGAGGAAAAGAAACCAACCGGGATTCCCTGAGTAGCTGCGAGCGAAACGGGAGAAGCTCAGCACGTAGGGATGGCGGCCCGCCCGTCTATCCGATTCCGTGTACTGGTACGTCTCACTATCCGTCTTCCTAGTGGTATTCAAGTCCAACTTGAACGTGGCTTTGAACCCATAGAGGGTGATAGGCCCGTAGAACACCACCCAGCGGTTGATGGACCGAGCGTACCATGGAGTCGTGTTGCTTGATAGTGCAGCACAAAGTGGGAGGTAAACTCCTTCTAAAGCTAAATACGACCATGAGACCGATAGTAAACAAGTACCGTGTGGGAAAGTTGAAAAGCACTCTGAATAGAGAGTCAAATAGTACGTGAAACTGCCGAGGGTGTAAAGCCTGTTGAACTCGATTATCCGTAGTTCCTGGCATCGTGGGTT |
| *Culex pipiens pallens* | CATTTTTGGGCTAATGGGGGGGGTTTATCTTATCGCTATCGATCGCATGCTCGTGTGTTGGGTTGTTCTGTTCTGTGGACGCGGTACCGCGACGACGCCGGATGACCGAACGAACGTGTGACGAGACGTGCGTGAGAGATGCTACATCGGCGCCCAAGCCAGGTTGGTGTGTGGGATGTATTCTCACCGTCGTCGTCGTCCGTTCAGTCGCGTGCGTGCGCACCACTGTGGCCGTCCGCCGAACGAAAACCCCGAGCCGCTGAGCGTCTTGAATGTTTTGCCAGCCACCGAAGGCAGCAAAACACCATTCTGCGTGCGTGTGTGCGCACAGTTGAATAGATAAATATAGGCACTCAAAAATGTGTACATCGTACTGTTGTACGATGTGCAATATGCGTTCAACTTGTCGGTGTTCATGTGTCCTGCAGTTCACATTCTGACGCGCATTTATCTGCGGTCTTCATCGATCCACGAGCCGAGTGATCCCCTGCCTAGGGTTATAA |
| *Anopheles anthropophagus* | GTCGTGCATAAAGGTGTAAGAGAGATCTCGTCGATCGCTTGCATCTCGGAACTTGTGTTGAAAGGCCGCGAAGACAGACAAGTAGTAAACAGCAGCAGATGTGTTCCCGCGATTGGCGGAAGTTCTAGGCAGGCGCGCCCTGACGCTGTGTGTAGATGGAGCAGGTGTCTTCCTCATCTATTTTTATTTTAAAAATTGAGGTAAGATTTCCAACGTTTCTTCGAGATAGTGGAATGGGCTGCAAGAGACTGGAATCGGAAGTTGAACAACGGAACACTCTATTAGCAAACACTACCCAGA |
| *Aedes albopictus* | CGGGGATGTGCGGTGCGGTGTCGTGGATTAGGCGCGTGCGCGGGAGCGCACGCGCGGCCCGGCGGGGCTTTTTCCGACGACACACAACGCCACCCGCAACGACGAACCACACACGGCAGCGGGGGAACAGTCACCTGAAACTCTCATGGGGCCCCTTCCCTTCACCCCATCCAATCCCCTAGCCCCAGGATTTTTTCATAAGGCCTCAAAAAAGGGGGAACAAA |
| *Armigeres subalbatus* | AAACCGACCCATCACCACCATCATCATGGCCCTGTGTGTGTGTGTGTGTGTGTGTGTGTCTTTGTTGTGTATTCCATCATCCCATATGTAATCCTCCTATGTAGGCCTCAAATAATGTGTGACTAA |
| *Aedes aegypti* | CAATCTGCTCGCTTGTGTTGTATTCCATCATTCACTAACTAACTAGCTAACTCTCTATAGTAGGCCTCAAATAATGTGTGACTAAA |
