# Supplementary material for: Real-time dynamic monitoring and multiplex PCR identification of vector mosquitoes in Zhejiang, China
Source: PLoS Negl Trop Dis. 2025 May 27;19(5):e0013129. doi: 10.1371/journal.pntd.0013129 (PMC12140415; doi:10.1371/journal.pntd.0013129)
Supplement: S3 File — (DOCX) [file pntd.0013129.s003.docx]

**The sequencing results of the cox1 gene of 106 field mosquito samples.**

> Sample1

GGGGCTTGAGCTGGAATAGTTGGAACTTCTTTAAGTTTACTAATTCGAGCAGAATTAAGTCAACCAGGTGTATTTATTGGAAATGATCAAATTTATAATGTTATTGTAACTGCTCATGCTTTTATTATAATTTTTTTTATAGTAATACCAATCATAATTGGAGGATTTGGAAATTGATTAGTTCCTTTAATGTTAGGAGCTCCAGATATGGCCTTTCCTCGAATAAATAATATAAGTTTTTGAATACTACCTCCTTCATTGACACTACTGCTTTCAAGTAGTTTAGTAGAAAATGGAGCTGGGACTGGATGAACAGTGTATCCCCCTCTTTCATCTGGAACAGCTCATGCTGGAGCTTCAGTAGACTTAGCTATTTTTTCTTTACATTTAGCAGGAATTTCATCAATTTTAGGTGCAGTAAATTTTATTACAACAGTAATTAATATACGATCTTCAGGAATTACTCTTGATCGAATACCTTTATTTGTTTGATCAGTAGTAATTACTGCAGTTTTATTACTTCTTTCTTTACCTGTTTTAGCTGGTGCTATTACTATGTTATTAACAGATCGAAATTTAAATACTTCATTCTTTGATCCAATTGGAGGAGGAGATCCAATT

> Sample 2

GGGGCTTGAGCTGGAATAGTTGGAACTTCTTTAAGTTTACTAATTCGAGCAGAATTAAGTCAACCAGGTGTATTTATTGGAAATGATCAAATTTATAATGTTATTGTAACTGCTCATGCTTTTATTATAATTTTTTTTATAGTAATACCAATCATAATTGGAGGATTTGGAAATTGATTAGTTCCTTTAATGTTAGGAGCTCCAGATATAGCCTTTCCTCGAATAAATAATATAAGTTTTTGAATACTACCTCCTTCATTGACACTACTACTTTCAAGTAGTTTAGTAGAAAATGGAGCTGGGACTGGATGAACAGTGTATCCCCCTCTTTCATCTGGAACAGCTCATGCTGGAGCTTCAGTAGACTTAGCTATTTTTTCTTTACATTTAGCAGGAATTTCATCAATTTTAGGTGCAGTAAATTTTATTACAACAGTAATTAATATACGATCTTCAGGAATTACTCTTGATCGAATACCTTTATTTGTTTGATCAGTAGTAATTACTGCAGTTTTATTACTTCTTTCTTTACCTGTTTTAGCTGGTGCTATTACTATGTTATTAACAGATCGAAATTTAAATACTTCATTCTTTGATCCAATTGG

> Sample 3

ATTTTATTTTTGGGGCTTGAGCTGGAATAGTTGGAACTTCTTTAAGTTTACTAATTCGAGCAGAATTAAGTCAACCAGGTGTATTTATTGGAAATGATCAAATTTATAATGTTATTGTAACTGCTCATGCTTTTATTATAATTTTTTTTATAGTAATACCAATCATAATTGGAGGATTTGGAAATTGATTAGTTCCTTTAATGTTAGGAGCTCCAGATATGGCCTTTCCTCGAATAAATAATATAAGTTTTTGAATACTACCTCCTTCATTGACACTACTACTTTCAAGTAGTTTAGTAGAAAATGGGGCTGGGACTGGATGAACAGTGTATCCCCCTCTTTCATCTGGAACAGCTCATGCTGGAGCTTCAGTAGACTTAGCTATTTTTTCTTTACATTTAGCAGGAATTTCATCAATTTTAGGTGCAGTAAATTTTATTACAACAGTAATTAATATACGATCTTCAGGAATTACTCTTGATCGAATACCTTTATTTGTTTGATCAGTAGTAATTACTGCAGTTTTATTACTTCTTTCTTTACCTGTTTTAGCTGGTGCTATTACTATGTTATTAACAGATCGAAATTTAAATACTTCATTCTTTGATCCAATTGGAGGAGGAGATCCAATTTTA

> Sample 4

GGAGCTTGAGCTGGAATAGTTGGAACTTCTTTAAGTTTACTAATTCGAGCAGAATTAAGTCAACCAGGTGTATTTATTGGAAATGATCAAATTTATAATGTTATTGTAACTGCTCATGCTTTTATTATAATTTTTTTTATAGTAATACCAATCATAATTGGAGGATTTGGAAATTGATTAGTTCCTTTAATGTTAGGAGCTCCAGATATGGCCTTTCCTCGAATAAATAATATAAGTTTTTGAATACTACCTCCTTCATTGACACTACTACTTTCAAGTAGTTTAGTAGAAAATGGAGCTGGGACTGGATGAACAGTGTATCCCCCTCTTTCATCTGGAACAGCTCATGCTGGAGCTTCAGTAGACTTAGCTATTTTTTCTTTACATTTAGCAGGAATTTCATCAATTTTAGGTGCAGTAAATTTTATTACAACAGTAATTAATATACGATCTTCAGGAATTACTCTTGATCGAATACCTTTATTTGTTTGATCAGTAGTAATTACTGCAGTTTTATTACTTCTTTCTTTACCTGTTTTAGCTGGTGCTATTACTATGTTATTAACAGATCGAAATTTAAATACTTCATTCTTTGATCCAATTGGAGGAGGAGATC

> Sample5

GGGGCTTGAGCTGGAATAGTTGGAACTTCTTTAAGTTTACTAATTCGAGCAGAATTAAGTCAACCAGGTGTATTTATTGGAAATGATCAAATTTATAATGTTATTGTAACTGCTCATGCTTTTATTATAATTTTTTTTATAGTAATACCAATCATAATTGGAGGATTTGGAAATTGATTAGTTCCTTTAATGTTAGGAGCTCCAGATATGGCCTTTCCTCGAATAAATAATATAAGTTTTTGAATACTACCTCCTTCATTGACACTACTACTTTCAAGTAGTTTAGTAGAAAATGGGGCTGGGACTGGATGAACAGTGTATCCCCCTCTTTCATCTGGAACAGCTCATGCTGGAGCTTCAGTAGACTTAGCTATTTTTTCTTTACATTTAGCAGGAATTTCATCAATTTTAGGTGCAGTAAATTTTATTACAACAGTAATTAATATACGATCTTCAGGAATTACTCTTGATCGAATACCTTTATTTATTTGATCAGTAGTAATTACTGCAGTTTTATTACTTCTTTCTTTACCTGTTTTAGCTGGTGCTATTACTATGTTATTAACAGATCGAAATTTAAATACTTCATTCTTTGATCCAATTGGAGGAGGAGA

> Sample6

GGCTTGAGCTGGAATAGTTGGAACTTCTTTAAGTTTACTAATTCGAGCAGAATTAAGTCAACCAGGTGTATTTATTGGAAATGATCAAATTTATAATGTTATTGTAACTGCTCATGCTTTTATTATAATTTTTTTTATAGTAATACCAATCATAATTGGAGGATTTGGAAATTGATTAGTTCCTTTAATGTTAGGAGCTCCAGATATGGCCTTTCCTCGAATAAATAATATAAGTTTTTGAATACTACCTCCTTCATTGACACTACTACTTTCAAGTAGTTTAGTAGAAAATGGAGCTGGGACTGGATGAACAGTGTATCCCCCTCTTTCATCTGGAACAGCTCATGCTGGAGCTTCAGTAGACTTAGCTATTTTTTCTTTACATTTAGCAGGAATTTCATCAATTTTAGGTGCAGTAAATTTTATTACAACAGTAATTAATATACGATCTTCAGGAATTACTCTTGATCGAATACCTTTATTTGTTTGATCAGTAGTAATTACTGCAGTTTTATTACTTCTTTCTTTACCTGTTTTAGCTGGTGCTATTACTATGTTATTAACAGATCGAAATTTAAATACTTCATTCTTTGATCCAATTGGAGGAGG

> Sample7

GGGGCCTTTGAGCTGGATAGTTGGAACTTCTTTAAGTTTACTAATTCGAGCAGAATTAAGTCAACCAGGTGTATTTATTGGAAATGATCAAATTTATAATGTTATTGTAACTGCTCATGCTTTTATTATAATTTTTTTTATAGTAATACCAATCATAATTGGAGGATTTGGAAATTGATTAGTTCCTTTAATGTTAGGAGCTCCAGATATGGCCTTTCCTCGAATAAATAATATAAGTTTTTGAATACTACCTCCTTCATTGACACTACTACTTTCAAGTAGTTTAGTAGAAAATGGAGCTGGGACTGGATGAACAGTGTATCCCCCTCTTTCATCTGGAACAGCTCATGCTGGAGCTTCAGTAGACTTAGCTATTTTTTCTTTACATTTAGCAGGAATTTCATCAATTTTAGGTGCAGTAAATTTTATTACAACAGTAATTAATATACGATCTTCAGGAATTACTCTTGATCGAATACCTTTATTTGTTTGATCAGTAGTAATTACTGCAGTTTTATTACTTCTTTCTTTACCTGTTTTAGCTGGTGCTATTACTATGTTATTAACAGATCGAAATTTAAATACTTCATTCTTTGATCCAATTGGAGGAGGAG

> Sample 8

TTTATTTTTGGGGCTTGAGCTGGAATAGTTGGAACTTCTTTAAGTTTACTAATTCGAGCAGAATTAAGTCAACCAGGTGTATTTATTGGAAATGATCAAATTTATAATGTTATTGTAACTGCTCATGCTTTTATTATAATTTTTTTTATAGTAATACCAATCATAATTGGAGGATTTGGAAATTGATTAGTTCCTTTAATGTTAGGAGCTCCAGATATGGCCTTTCCTCGAATAAATAATATAAGTTTTTGAATACTACCTCCTTCATTGACACTACTACTTTCAAGTAGTTTAGTAGAAAATGGGGCTGGGACTGGATGAACAGTGTATCCCCCTCTTTCATCTGGAACAGCTCATGCTGGAGCTTCAGTAGACTTAGCTATTTTTTCTTTACATTTAGCAGGAATTTCATCAATTTTAGGTGCAGTAAATTTTATTACAACAGTAATTAATATACGATCTTCAGGAATTACTCTTGATCGAATACCTTTATTTGTTTGATCAGTAGTAATTACTGCAGTTTTATTACTTCTTTCTTTACCTGTTTTAGCTGGTGCTATTACTATGTTATTAACAGATCGAAATTTAAATACTTCATTCTTTGATCCAATTGGAGGAGGAGATCCAATTTTATATCAA

> Sample 9

ATTTTATTTTTGGGGCTTGAGCTGGAATAGTTGGAACTTCTTTAAGTTTACTAATTCGAGCAGAATTAAGTCAACCAGGTGTATTTATTGGAAATGATCAAATTTATAATGTTATTGTAACTGCTCATGCTTTTATTATAATTTTTTTTATAGTAATACCAATCATAATTGGAGGATTTGGAAATTGATTAGTTCCTTTAATGTTAGGAGCTCCAGATATGGCCTTTCCTCGAATAAATAATATAAGTTTTTGAATACTACCTCCTTCATTGACACTACTACTTTCAAGTAGTTTAGTAGAAAATGGAGCTGGGACTGGATGAACAGTGTATCCCCCTCTTTCATCTGGAACAGCTCATGCTGGAGCTTCAGTAGACTTAGCTATTTTTTCTTTACATTTAGCAGGAATTTCATCAATTTTAGGTGCAGTAAATTTTATTACAACAGTAATTAATATACGATCTTCAGGAATTACTCTTGATCGAATACCTTTATTTGTTTGATCAGTAGTAATTACTGCAGTTTTATTACTTCTTTCTTTACCTGTTTTAGCTGGTGCTATTACTATGTTATTAACAGATCGAAATTTAAATACTTCATTCTTTGATCCAATTGGAGGAGGAGATCCAATTTTATATCCAACAT

> Sample 10

ATTATATTTTATTTTTGGGGCTTGAGCTGGAATAGTTGGAACTTCTTTAAGTTTACTAATTCGAGCAGAATTAAGTCAACCAGGTGTATTTATTGGAAATGATCAAATTTATAATGTTATTGTAACTGCTCATGCTTTTATTATAATTTTTTTTATAGTAATACCAATCATAATTGGAGGATTTGGAAATTGATTAGTTCCTTTAATGTTAGGAGCTCCAGATATGGCCTTTCCTCGAATAAATAATATAAGTTTTTGAATACTACCTCCTTCATTGACACTACTACTTTCAAGTAGTTTAGTAGAAAATGGGGCTGGGACTGGATGAACAGTGTATCCCCCTCTTTCATCTGGAACAGCTCATGCTGGAGCTTCAGTAGACTTAGCTATTTTTTCTTTACATTTAGCAGGAATTTCATCAATTTTAGGTGCAGTAAATTTTATTACAACAGTAATTAATATACGATCTTCAGGAATTACTCTTGATCGAATACCTTTATTTGTTTGATCAGTAGTAATTACTGCAGTTTTATTACTTCTTTCTTTACCTGTTTTAGCTGGTGCTATTACTATGTTATTAACAGATCGAAATTTAAATACTTCATTCTTTGATCCAATTGGAGGAGGAGATCCAATTTTATATCAAC

> Sample 11

ATTTTATTTTTGGGGCTTGAGCTGGAATAGTTGGAACTTCTTTAAGTTTACTAATTCGAGCAGAATTAAGTCAACCAGGTGTATTTATTGGAAATGATCAAATTTATAATGTTATTGTAACTGCTCATGCTTTTATTATAATTTTTTTTATAGTAATACCAATCATAATTGGAGGATTTGGAAATTGATTAGTTCCTTTAATGTTAGGAGCTCCAGATATGGCCTTTCCTCGAATAAATAATATAAGTTTTTGAATACTACCTCCTTCATTGACACTACTACTTTCAAGTAGTTTAGTAGAAAATGGAGCTGGGACTGGATGAACAGTGTATCCCCCTCTTTCATCTGGAACAGCTCATGCTGGAGCTTCAGTAGACTTAGCTATTTTTTCTTTACATTTAGCAGGAATTTCATCAATTTTAGGTGCAGTAAATTTTATTACAACAGTAATTAATATACGATCTTCAGGAATTACTCTTGATCGAATACCTTTATTTGTTTGATCAGTAGTAATTACTGCAGTTTTATTACTTCTTTCTTTACCTGTTTTAGCTGGTGCTATTACTATGTTATTAACAGATCGAAATTTAAATACTTCATTCTTTGATCCAATTGGAGGAGGAGATCCAATTTTATATCAACATT

> Sample 12

TTTTATTTTTGGGGCTTGAGCTGGAATAGTTGGAACTTCTTTAAGTTTACTAATTCGAGCAGAATTAAGTCAACCAGGTGTATTTATTGGAAATGATCAAATTTATAATGTTATTGTAACTGCTCATGCTTTTATTATAATTTTTTTTATAGTAATACCAATCATAATTGGAGGATTTGGAAATTGATTAGTTCCTTTAATGTTAGGAGCTCCAGATATGGCCTTTCCTCGAATAAATAATATAAGTTTTTGAATACTACCTCCTTCATTGACACTACTACTTTCAAGTAGTTTAGTAGAAAATGGAGCTGGGACTGGATGAACAGTGTATCCCCCTCTTTCATCTGGAACAGCTCATGCTGGAGCTTCAGTAGACTTAGCTATTTTTTCTTTACATTTAGCAGGAATTTCATCAATTTTAGGTGCAGTAAATTTTATTACAACAGTAATTAATATACGATCTTCAGGAATTACTCTTGATCGAATACCTTTATTTGTTTGATCAGTAGTAATTACTGCAGTTTTATTACTTCTTTCTTTACCTGTTTTAGCTGGTGCTATTACTATGTTATTAACAGATCGAAATTTAAATACTTCATTCTTTGATCCAATTGGAGGAGGAGATCCAATTTTATATCAAC

> Sample 13

TTTTTGGGGCTTGAGCTGGAATAGTTGGAACTTCTTTAAGTTTACTAATTCGAGCAGAATTAAGTCAACCAGGTGTATTTATTGGAAATGATCAAATTTATAATGTTATTGTAACTGCTCATGCTTTTATTATAATTTTTTTTATAGTAATACCAATCATAATTGGAGGATTTGGAAATTGATTAGTTCCTTTAATGTTAGGAGCTCCAGATATGGCCTTTCCTCGAATAAATAATATAAGTTTTTGAATACTACCTCCTTCATTGACACTACTACTTTCAAGTAGTTTAGTAGAAAATGGGGCTGGGACTGGATGAACAGTGTATCCCCCTCTTTCATCTGGAACAGCTCATGCTGGAGCTTCAGTAGACTTAGCTATTTTTTCTTTACATTTAGCAGGAATTTCATCAATTTTAGGTGCAGTAAATTTTATTACAACAGTAATTAATATACGATCTTCAGGAATTACTCTTGATCGAATACCTTTATTTGTTTGATCAGTAGTAATTACTGCAGTTTTATTACTTCTTTCTTTACCTGTTTTAGCTGGTGCTATTACTATGTTATTAACAGATCGAAATTTAAATACTTCATTCTTTGATCCAATTGGAGGAGGAGATCCAATTTTATATCAACA

> Sample 14

TTTTATTTTTGGGGCTTGAGCTGGAATAGTTGGAACTTCTTTAAGTTTACTAATTCGAGCAGAATTAAGTCAACCAGGTGTATTTATTGGAAATGATCAAATTTATAATGTTATTGTAACTGCTCATGCTTTTATTATAATTTTTTTTATAGTAATACCAATCATAATTGGAGGATTTGGAAATTGATTAGTTCCTTTAATGTTAGGAGCTCCAGATATGGCCTTTCCTCGAATAAATAATATAAGTTTTTGAATACTACCTCCTTCATTGACACTACTACTTTCAAGTAGTTTAGTAGAAAATGGAGCTGGGACTGGATGAACAGTGTATCCCCCTCTTTCATCTGGAACAGCTCATGCTGGAGCTTCAGTAGACTTAGCTATTTTTTCTTTACATTTAGCAGGAATTTCATCAATTTTAGGTGCAGTAAATTTTATTACAACAGTAATTAATATACGATCTTCAGGAATTACTCTTGATCGAATACCTTTATTTGTTTGATCAGTAGTAATTACTGCAGTTTTATTACTTCTTTCTTTACCTGTTTTAGCTGGTGCTATTACTATGTTATTAACAGATCGAAATTTAAATACTTCATTCTTTGATCCAATTGGAGGAGGAGATCCAATTTTATATCAAC

> Sample 15

TCCTTTTATTTTTGGGGCTTGAGCTGGATAGTTGGAACTTCTTTAAGTTTACTAATTCGAGCAGAATTAAGTCAACCAGGTGTATTTATTGGAAATGATCAAATTTATAATGTTATTGTAACTGCTCATGCTTTTATTATAATTTTTTTTATAGTAATACCAATCATAATTGGAGGATTTGGAAATTGATTAGTTCCTTTAATGTTAGGAGCTCCAGATATGGCCTTTCCTCGAATAAATAATATAAGTTTTTGAATACTACCTCCTTCATTGACACTACTACTTTCAAGTAGTTTAGTAGAAAATGGAGCTGGGACTGGATGAACAGTGTATCCCCCTCTTTCATCTGGAACAGCTCATGCTGGAGCTTCAGTAGACTTAGCTATTTTTTCTTTACATTTAGCAGGAATTTCATCAATTTTAGGTGCAGTAAATTTTATTACAACAGTAATTAATATACGATCTTCAGGAATTACTCTTGATCGAATACCTTTATTTGTTTGATCAGTAGTAATTACTGCAGTTTTATTACTTCTTTCTTTACCTGTTTTAGCTGGTGCTATTACTATGTTATTAACAGATCGAAATTTAAATACTTCATTCTTTGATCCAATTGGAGGAGGAGATCCAATTTTATATCAACATTTATTTTGATTTTTTGGTCACCCTGGAAGTTTA

> Sample16

TTGAGGGGCTTGAGCTGGAATAGTTGGAACTTCTTTAAGTTTACTAATTCGAGCAGAATTAAGTCAACCAGGTGTATTTATTGGAAATGATCAAATTTATAATGTTATTGTAACTGCTCATGCTTTTATTATAATTTTTTTTATAGTAATACCAATCATAATTGGAGGATTTGGAAATTGATTAGTTCCTTTAATGTTAGGAGCTCCAGATATGGCCTTTCCTCGAATAAATAATATAAGTTTTTGAATACTACCTCCTTCATTGACACTACTACTTTCAAGTAGTTTAGTAGAAAATGGGGCTGGGACTGGATGAACAGTGTATCCCCCTCTTTCATCTGGAACAGCTCATGCTGGAGCTTCAGTAGACTTAGCTATTTTTTCTTTACATTTAGCAGGAATTTCATCAATTTTAGGTGCAGTAAATTTTATTACAACAGTAATTAATATACGATCTTCAGGAATTACTCTTGATCGAATACCTTTATTTGTTTGATCAGTAGTAATTACTGCAGTTTTATTACTTCTTTCTTTACCTGTTTTAGCTGGTGCTATTACTATGTTATTAACAGATCGAAATTTAAATACTTCATTCTTTGATCCAATTGGAGGAGGAGATCCAATTTTATATCAACATTTATTTTGATTTTTTGGTCACCCTGGAAGTTTA

> Sample 17

TACTTTTTGGGGCTTGAGCTGGAATAGTTGGAACTTCTTTAAGTTTACTAATTCGAGCAGAATTAAGTCAACCAGGTGTATTTATTGGAAATGATCAAATTTATAATGTTATTGTAACTGCTCATGCTTTTATTATAATTTTTTTTATAGTAATACCAATCATAATTGGAGGATTTGGAAATTGATTAGTTCCTTTAATGTTAGGAGCTCCAGATATAGCCTTTCCTCGAATAAATAATATAAGTTTTTGAATACTACCTCCTTCATTGACACTACTACTTTCAAGTAGTTTAGTAGAAAATGGAGCTGGGACTGGATGAACAGTGTATCCCCCTCTTTCATCTGGAACAGCTCATGCTGGAGCTTCAGTAGACTTAGCTATTTTTTCTTTACATTTAGCAGGAATTTCATCAATTTTAGGTGCAGTAAATTTTATTACAACAGTAATTAATATACGATCTTCAGGAATTACTCTTGATCGAATACCTTTATTTGTTTGATCAGTAGTAATTACTGCAGTTTTATTACTTCTTTCTTTACCTGTTTTAGCTGGTGCTATTACTATGTTATTAACAGATCGAAATTTAAATACTTCATTCTTTGATCCAATTGGAGGAGGAGATCCAATTTTATATCAACATTTATTTTGATTTTTTGGTCACCTTGGAAAGTTTAA

> Sample 18

TTTGTGGGCTTGAGCTGGAATAGTTGGAACTTCTTTAAGTTTACTAATTCGAGCAGAATTAAGTCAACCAGGTGTATTTATTGGAAATGATCAAATTTATAATGTTATTGTAACTGCTCATGCTTTTATTATAATTTTTTTTATAGTAATACCAATCATAATTGGAGGATTTGGAAATTGATTAGTTCCTTTAATGTTAGGAGCTCCAGATATGGCCTTTCCTCGAATAAATAATATAAGTTTTTGAATACTACCTCCTTCATTGACACTACTACTTTCAAGTAGTTTAGTAGAAAATGGGGCTGGGACTGGATGAACAGTGTATCCCCCTCTTTCATCTGGAACAGCTCATGCTGGAGCTTCAGTAGACTTAGCTATTTTTTCTTTACATTTAGCAGGAATTTCATCAATTTTAGGTGCAGTAAATTTTATTACAACAGTAATTAATATACGATCTTCAGGAATTACTCTTGATCGAATACCTTTATTTATTTGATCAGTAGTAATTACTGCAGTTTTATTACTTCTTTCTTTACCTGTTTTAGCTGGTGCTATTACTATGTTATTAACAGATCGAAATTTAAATACTTCATTCTTTGATCCAATTGGAGGAGGAGATCCAATTTTATATCAACATTTATTTTGATTTTTTGGTCACCCGGGAAGTTTAA

> Sample19

CAACCGCGACTTTTGGGGCTTGAGCTGGATAGTTGGAACTTCTTTAAGTTTACTAATTCGAGCAGAATTAAGTCAACCAGGTGTATTTATTGGAAATGATCAAATTTATAATGTTATTGTAACTGCTCATGCTTTTATTATAATTTTTTTTATAGTAATACCAATCATAATTGGAGGATTTGGAAATTGATTAGTTCCTTTAATGTTAGGAGCTCCAGATATGGCCTTTCCTCGAATAAATAATATAAGTTTTTGAATACTACCTCCTTCATTGACACTACTACTTTCAAGTAGTTTAGTAGAAAATGGAGCTGGGACTGGATGAACAGTGTATCCCCCTCTTTCATCTGGAACAGCTCATGCTGGAGCTTCAGTAGACTTAGCTATTTTTTCTTTACATTTAGCAGGAATTTCATCAATTTTAGGTGCAGTAAATTTTATTACAACAGTAATTAATATACGATCTTCAGGAATTACTCTTGATCGAATACCTTTATTTGTTTGATCAGTAGTAATTACTGCAGTTTTATTACTTCTTTCTTTACCTGTTTTAGCTGGTGCTATTACTATGTTATTAACAGATCGAAATTTAAATACTTCATTCTTTGATCCAATTGGAGGAGGAGATCCAATTTTATATCAACATTTATTTTGATTTTTTGGTCACCCTGGAAGTTTA

> Sample 20

GCGGGGGCTTGAGCTGGATAGTTGGAACTTCTTTAAGTTTACTAATTCGAGCAGAATTAAGTCAACCAGGTGTATTTATTGGAAATGATCAAATTTATAATGTTATTGTAACTGCTCATGCTTTTATTATAATTTTTTTTATAGTAATACCAATCATAATTGGAGGATTTGGAAATTGATTAGTTCCTTTAATGTTAGGAGCTCCAGATATGGCCTTTCCTCGAATAAATAATATAAGTTTTTGAATACTACCTCCTTCATTGACACTACTACTTTCAAGTAGTTTAGTAGAAAATGGGGCTGGGACTGGATGAACAGTGTATCCCCCTCTTTCATCTGGAACAGCTCATGCTGGAGCTTCAGTAGACTTAGCTATTTTTTCTTTACATTTAGCAGGAATTTCATCAATTTTAGGTGCAGTAAATTTTATTACAACAGTAATTAATATACGATCTTCAGGAATTACTCTTGATCGAATACCTTTATTTGTTTGATCAGTAGTAATTACTGCAGTTTTATTACTTCTTTCTTTACCTGTTTTAGCTGGTGCTATTACTATGTTATTAACAGATCGAAATTTAAATACTTCATTCTTTGATCCAATTGGAGGAGGAGATCCAATTTTATATCAACATTTATTTTGATTTTTTGGTCACCCTGGAAGTTTA

> Sample 21

AAAGGGGGCTTGAGCTGGAATAGTTGGAACTTCTTTAAGTTTACTAATTCGAGCAGAATTAAGTCAACCAGGTGTATTTATTGGAAATGATCAAATTTATAATGTTATTGTAACTGCTCATGCTTTTATTATAATTTTTTTTATAGTAATACCAATCATAATTGGAGGATTTGGAAATTGATTAGTTCCTTTAATGTTAGGAGCTCCAGATATGGCCTTTCCTCGAATAAATAATATAAGTTTTTGAATACTACCTCCTTCATTGACACTACTACTTTCAAGTAGTTTAGTAGAAAATGGAGCTGGGACTGGATGAACAGTGTATCCCCCTCTTTCATCTGGAACAGCTCATGCTGGAGCTTCAGTAGACTTAGCTATTTTTTCTTTACATTTAGCAGGAATTTCATCAATTTTAGGTGCAGTAAATTTTATTACAACAGTAATTAATATACGATCTTCAGGAATTACTCTTGATCGAATACCTTTATTTGTTTGATCAGTAGTAATTACTGCAGTTTTATTACTTCTTTCTTTACCTGTTTTAGCTGGTGCTATTACTATGTTATTAACAGATCGAAATTTAAATACTTCATTCTTTGATCCAATTGGAGGAGGAGATCCAATTTTATATCAACATTTATTTTGATTTTTTGGTCACCCTGGAAGTTTAA

> Sample 22

AAAAATTTATTTTTGGGGCTTGAGCTGGAATAGTTGGAACTTCTTTAAGTTTACTAATTCGAGCAGAATTAAGTCAACCAGGTGTATTTATTGGAAATGATCAAATTTATAATGTTATTGTAACTGCTCATGCTTTTATTATAATTTTTTTTATAGTAATACCAATCATAATTGGAGGATTTGGAAATTGATTAGTTCCTTTAATGTTAGGAGCTCCAGATATAGCCTTTCCTCGAATAAATAATATAAGTTTTTGAATACTACCTCCTTCATTGACACTACTACTTTCAAGTAGTTTAGTAGAAAATGGAGCTGGGACTGGATGAACAGTGTATCCCCCTCTTTCATCTGGAACAGCTCATGCTGGAGCTTCAGTAGACTTAGCTATTTTTTCTTTACATTTAGCAGGAATTTCATCAATTTTAGGTGCAGTAAATTTTATTACAACAGTAATTAATATACGATCTTCAGGAATTACTCTTGATCGAATACCTTTATTTGTTTGATCAGTAGTAATTACTGCAGTTTTATTACTTCTTTCTTTACCTGTTTTAGCTGGTGCTATTACTATGTTATTAACAGATCGAAATTTAAATACTTCATTCTTTGATCCAATTGGAGGAGGAGATCCAATTTTATATCAACATTTATTTTGATTTTTTGGTCACCCTGGAAAGTTTA

> Sample 23

AACCCATAGCCTGGGGCTTGAGCTGGATAGTTGGAACTTCTTTAAGTTTACTAATTCGAGCAGAATTAAGTCAACCAGGTGTATTTATTGGAAATGATCAAATTTATAATGTTATTGTAACTGCTCATGCTTTTATTATAATTTTTTTTATAGTAATACCAATCATAATTGGAGGATTTGGAAATTGATTAGTTCCTTTAATGTTAGGAGCTCCAGATATAGCCTTTCCTCGAATAAATAATATAAGTTTTTGAATACTACCTCCTTCATTGACACTACTACTTTCAAGTAGTTTAGTAGAAAATGGAGCTGGGACTGGATGAACAGTGTATCCCCCTCTTTCATCTGGAACAGCTCATGCTGGAGCTTCAGTAGACTTAGCTATTTTTTCTTTACATTTAGCAGGAATTTCATCAATTTTAGGTGCAGTAAATTTTATTACAACAGTAATTAATATACGATCTTCAGGAATTACTCTTGATCGAATACCTTTATTTGTTTGATCAGTAGTAATTACTGCAGTTTTATTACTTCTTTCTTTACCTGTTTTAGCTGGTGCTATTACTATGTTATTAACAGATCGAAATTTAAATACTTCATTCTTTGATCCAATTGGAGGAGGAGATCCAATTTTATATCAACATTTATTTTGATTTTTTGGTCACCGGGAAAGTTTTA

> Sample24

ACGGTTGGGGGCTTGAGCTGGATAGTTGGAACTTCTTTAAGTTTACTAATTCGAGCAGAATTAAGTCAACCAGGTGTATTTATTGGAAATGATCAAATTTATAATGTTATTGTAACTGCTCATGCTTTTATTATAATTTTTTTTATAGTAATACCAATCATAATTGGAGGATTTGGAAATTGATTAGTTCCTTTAATGTTAGGAGCTCCAGATATGGCCTTTCCTCGAATAAATAATATAAGTTTTTGAATACTACCTCCTTCATTGACACTACTACTTTCAAGTAGTTTAGTAGAAAATGGAGCTGGGACTGGATGAACAGTGTATCCCCCTCTTTCATCTGGAACAGCTCATGCTGGAGCTTCAGTAGACTTAGCTATTTTTTCTTTACATTTAGCAGGAATTTCATCAATTTTAGGTGCAGTAAATTTTATTACAACAGTAATTAATATACGATCTTCAGGAATTACTCTTGATCGAATACCTTTATTTGTTTGATCAGTAGTAATTACTGCAGTTTTATTACTTCTTTCTTTACCTGTTTTAGCTGGTGCTATTACTATGTTATTAACAGATCGAAATTTAAATACTTCATTCTTTGATCCAATTGGAGGAGGAGATCCAATTTTATATCAACATTTATTTTGATTTTTTGGTCACCCTGAAGTTTAA

> Sample 25

ACCCCCCCTTTTGGGGCTTGAGCTGGAATAGTTGGAACTTCTTTAAGTTTACTAATTCGAGCAGAATTAAGTCAACCAGGTGTATTTATTGGAAATGATCAAATTTATAATGTTATTGTAACTGCTCATGCTTTTATTATAATTTTTTTTATAGTAATACCAATCATAATTGGAGGATTTGGAAATTGATTAGTTCCTTTAATGTTAGGAGCTCCAGATATAGCCTTTCCTCGAATAAATAATATAAGTTTTTGAATACTACCTCCTTCATTGACACTACTACTTTCAAGTAGTTTAGTAGAAAATGGAGCTGGGACTGGATGAACAGTGTATCCCCCTCTTTCATCTGGAACAGCTCATGCTGGAGCTTCAGTAGACTTAGCTATTTTTTCTTTACATTTAGCAGGAATTTCATCAATTTTAGGTGCAGTAAATTTTATTACAACAGTAATTAATATACGATCTTCAGGAATTACTCTTGATCGAATACCTTTATTTGTTTGATCAGTAGTAATTACTGCAGTTTTATTACTTCTTTCTTTACCTGTTTTAGCTGGTGCTATTACTATGTTATTAACAGATCGAAATTTAAATACTTCATTCTTTGATCCAATTGGAGGAGGAGATCCAATTTTATATCAACATTTATTTTGATTTTTTGGTCACCTGGGAAGTTTAA

> Sample 26

TTGTGGGGCTTGAGCTGGATAGTTGGAACTTCTTTAAGTTTACTAATTCGAGCAGAATTAAGTCAACCAGGTGTATTTATTGGAAATGATCAAATTTATAATGTTATTGTAACTGCTCATGCTTTTATTATAATTTTTTTTATAGTAATACCAATCATAATTGGAGGATTTGGAAATTGATTAGTTCCTTTAATGTTAGGAGCTCCAGATATGGCCTTTCCTCGAATAAATAATATAAGTTTTTGAATACTACCTCCTTCATTGACACTACTACTTTCAAGTAGTTTAGTAGAAAATGGAGCTGGGACTGGATGAACAGTGTATCCCCCTCTTTCATCTGGAACAGCTCATGCTGGAGCTTCAGTAGACTTAGCTATTTTTTCTTTACATTTAGCAGGAATTTCATCAATTTTAGGTGCAGTAAATTTTATTACAACAGTAATTAATATACGATCTTCAGGAATTACTCTTGATCGAATACCTTTATTTGTTTGATCAGTAGTAATTACTGCAGTTTTATTACTTCTTTCTTTACCTGTTTTAGCTGGTGCTATTACTATGTTATTAACAGATCGAAATTTAAATACTTCATTCTTTGATCCAATTGGAGGAGGAGATCCAATTTTATATCAACATTTATTTTGATTTTTTGGTCACCCTGGAAGTTTA

> Sample 27

TTGGGGGCTTGAGCTGGAATAGTTGGAACTTCTTTAAGTTTACTAATTCGAGCAGAATTAAGTCAACCAGGTGTATTTATTGGAAATGATCAAATTTATAATGTTATTGTAACTGCTCATGCTTTTATTATAATTTTTTTTATAGTAATACCAATCATAATTGGAGGATTTGGAAATTGATTAGTTCCTTTAATGTTAGGAGCTCCAGATATGGCCTTTCCTCGAATAAATAATATAAGTTTTTGAATACTACCTCCTTCATTGACACTACTACTTTCAAGTAGTTTAGTAGAAAATGGAGCTGGGACTGGATGAACAGTGTATCCCCCTCTTTCATCTGGAACAGCTCATGCTGGAGCTTCAGTAGACTTAGCTATTTTTTCTTTACATTTAGCAGGAATTTCATCAATTTTAGGTGCAGTAAATTTTATTACAACAGTAATTAATATACGATCTTCAGGAATTACTCTTGATCGAATACCTTTATTTGTTTGATCAGTAGTAATTACTGCAGTTTTATTACTTCTTTCTTTACCTGTTTTAGCTGGTGCTATTACTATGTTATTAACAGATCGAAATTTAAATACTTCATTCTTTGATCCAATTGGAGGAGGAGATCCAATTTTATATCAACATTTATTTTGATTTTTTGGTCACCCTGGAAGTTTAA

> Sample 28

TGGGGGCTTGAGCTGGAATAGTTGGAACTTCTTTAAGTTTACTAATTCGAGCAGAATTAAGTCAACCAGGTGTATTTATTGGAAATGATCAAATTTATAATGTTATTGTAACTGCTCATGCTTTTATTATAATTTTTTTTATAGTAATACCAATCATAATTGGAGGATTTGGAAATTGATTAGTTCCTTTAATGTTAGGAGCTCCAGATATGGCCTTTCCTCGAATAAATAATATAAGTTTTTGAATACTACCTCCTTCATTGACACTACTACTTTCAAGTAGTTTAGTAGAAAATGGAGCTGGGACTGGATGAACAGTGTATCCCCCTCTTTCATCTGGAACAGCTCATGCTGGAGCTTCAGTAGACTTAGCTATTTTTTCTTTACATTTAGCAGGAATTTCATCAATTTTAGGTGCAGTAAATTTTATTACAACAGTAATTAATATACGATCTTCAGGAATTACTCTTGATCGAATACCTTTATTTGTTTGATCAGTAGTAATTACTGCAGTTTTATTACTTCTTTCTTTACCTGTTTTAGCTGGTGCTATTACTATATTATTAACAGATCGAAATTTAAATACTTCATTCTTTGATCCAATTGGAGGAGGAGATCCAATTTTATATCAACATTTATTTTGATTTTTTGGTCACCCTGGAAGTTTA

> Sample 29

CTTGGGGGCTTGAGCTGGATAGTTGGAACTTCTTTAAGTTTACTAATTCGAGCAGAATTAAGTCAACCAGGTGTATTTATTGGAAATGATCAAATTTATAATGTTATTGTAACTGCTCATGCTTTTATTATAATTTTTTTTATAGTAATACCAATCATAATTGGAGGATTTGGAAATTGATTAGTTCCTTTAATGTTAGGAGCTCCAGATATGGCCTTTCCTCGAATAAATAATATAAGTTTTTGAATACTACCTCCTTCATTGACACTACTACTTTCAAGTAGTTTAGTAGAAAATGGAGCTGGGACTGGATGAACAGTGTATCCCCCTCTTTCATCTGGAACAGCTCATGCTGGAGCTTCAGTAGACTTAGCTATTTTTTCTTTACATTTAGCAGGAATTTCATCAATTTTAGGTGCAGTAAATTTTATTACAACAGTAATTAATATACGATCTTCAGGAATTACTCTTGATCGAATACCTTTATTTGTTTGATCAGTAGTAATTACTGCAGTTTTATTACTTCTTTCTTTACCTGTTTTAGCTGGTGCTATTACTATGTTATTAACAGATCGAAATTTAAATACTTCATTCTTTGACCCAATTGGAGGAGGAGATCCAATTTTATATCAACATTTATTTTGATTTTTTGGTCACCCTGGAAGTTTAA

> Sample 30

AAAATTTGGGGGAACTTCCTTTTAAGTTTACTAATTCGAGCAGAATTAAGTCAACCAGGTGTATTTATTGGAAATGATCAAATTTATAATGTTATTGTAACTGCTCATGCTTTTATTATAATTTTTTTTATAGTAATACCAATCATAATTGGAGGATTTGGAAATTGATTAGTTCCTTTAATGTTAGGAGCTCCAGATATGGCCTTTCCTCGAATAAATAATATAAGTTTTTGAATACTACCTCCTTCATTGACACTACTACTTTCAAGTAGTTTAGTAGAAAATGGAGCTGGGACTGGATGAACAGTGTATCCCCCTCTTTCATCTGGAACAGCTCATGCTGGAGCTTCAGTAGACTTAGCTATTTTTTCTTTACATTTAGCAGGAATTTCATCAATTTTAGGTGCAGTAAATTTTATTACAACAGTAATTAATATACGATCTTCAGGAATTACTCTTGATCGAATACCTTTATTTGTTTGATCAGTAGTAATTACTGCAGTTTTATTACTTCTTTCTTTACCTGTTTTAGCTGGTGCTATTACTATGTTATTAACAGATCGAAATTTAAATACTTCATTCTTTGATCCAATTGGAGGAGGAGATCCAATTTTATATCAACATTTATTTTGATTTTTTGGTCACCCTGAAGTTTAA

> Sample 31

CTTTTTTATTTTTGGGGCTTGAGCTGGAATAGTTGGAACTTCTTTAAGTTTACTAATTCGAGCAGAATTAAGTCAACCAGGTGTATTTATTGGAAATGATCAAATTTATAATGTTATTGTAACTGCTCATGCTTTTATTATAATTTTTTTTATAGTAATACCAATCATAATTGGAGGATTTGGAAATTGATTAGTTCCTTTAATGTTAGGAGCTCCAGATATGGCCTTTCCTCGAATAAATAATATAAGTTTTTGAATACTACCTCCTTCATTGACACTACTACTTTCAAGTAGTTTAGTAGAAAATGGAGCTGGGACTGGATGAACAGTGTATCCCCCTCTTTCATCTGGAACAGCTCATGCTGGAGCTTCAGTAGACTTAGCTATTTTTTCTTTACATTTAGCAGGAATTTCATCAATTTTAGGTGCAGTAAATTTTATTACAACAGTAATTAATATACGATCTTCAGGAATTACTCTTGATCGAATACCTTTATTTGTTTGATCAGTAGTAATTACTGCAGTTTTATTACTTCTTTCTTTACCTGTTTTAGCTGGTGCTATTACTATGTTATTAACAGATCGAAATTTAAATACTTCATTCTTTGATCCAATTGGAGGAGGAGATCCAATTTTATATCAACATTTATTTTGATTTTTTGGTCACCCTGAAGTTTAA

> Sample 32

TCCTTGAGCTGGATAGTTGGAACTTCTTTAAGTTTACTAATTCGAGCAGAATTAAGTCAACCAGGTGTATTTATTGGAAATGATCAAATTTATAATGTTATTGTAACTGCTCATGCTTTTATTATAATTTTTTTTATAGTAATACCAATCATAATTGGAGGATTTGGAAATTGATTAGTTCCTTTAATGTTAGGAGCTCCAGATATGGCCTTTCCTCGAATAAATAATATAAGTTTTTGAATACTACCTCCTTCATTGACACTACTACTTTCAAGTAGTTTAGTAGAAAATGGAGCTGGGACTGGATGAACAGTGTATCCCCCTCTTTCATCTGGGACAGCTCATGCTGGAGCTTCAGTAGACTTAGCTATTTTTTCTTTACATTTAGCAGGAATTTCATCAATTTTAGGTGCAGTAAATTTTATTACAACAGTAATTAATATACGATCTTCAGGAATTACTCTTGATCGAATACCTTTATTTGTTTGATCAGTAGTAATTACTGCAGTTTTATTACTTCTTTCTTTACCTGTTTTAGCTGGTGCTATTACTATGTTATTAACAGATCGAAATTTAAATACTTCATTCTTTGATCCAATTGGAGGAGGAGATCCAATTTTATATCAACATTTATTTTGATTTTTTGGTCACCCTGGAAGTTTAA

> Sample 33

CCGGCTTGGGAGCTGGATAGTTGGAACTTCTTTAAGTTTACTAATTCGAGCAGAATTAAGTCAACCAGGTGTATTTATTGGAAATGATCAAATTTATAATGTTATTGTAACTGCTCATGCTTTTATTATAATTTTTTTTATAGTAATACCAATCATAATTGGAGGATTTGGAAATTGATTAGTTCCTTTAATGTTAGGAGCTCCAGATATGGCCTTTCCTCGAATAAATAATATAAGTTTTTGAATACTACCTCCTTCATTGACACTACTACTTTCAAGTAGTTTAGTAGAAAATGGAGCTGGGACTGGATGAACAGTGTATCCCCCTCTTTCATCTGGAACAGCTCATGCTGGAGCTTCAGTAGACTTAGCTATTTTTTCTTTACATTTAGCAGGAATTTCATCAATTTTAGGTGCAGTAAATTTTATTACAACAGTAATTAATATACGATCTTCAGGAATTACTCTTGATCGAATACCTTTATTTGTTTGATCAGTAGTAATTACTGCAGTTTTATTACTTCTTTCTTTACCTGTTTTAGCTGGTGCTATTACTATGTTATTAACAGATCGAAATTTAAATACTTCATTCTTTGATCCAATTGGAGGAGGAGATCCAATTTTATATCAACATTTATTTTGATTTTTTGGTCACCCTGGAAGTTTA

> Sample 34

TGTGGGGCTTGAGCTGGAATAGTTGGAACTTCTTTAAGTTTACTAATTCGAGCAGAATTAAGTCAACCAGGTGTATTTATTGGAAATGATCAAATTTATAATGTTATTGCAACTGCTCATGCTTTTATTATAATTTTTTTTATAGTAATACCAATCATAATTGGAGGATTTGGAAATTGATTATTTCCTTTAATGTTAGGAGCTCCAGATATGGCCTTTCCTCTAATAAATAATATAAGTTTTTGAATACTACCTCCTTCATTGACACTACTACTTTCAAGTAGTTTAGTAGAAAATGGAGCTGGGACTGGATGAACAGGGTATCCCCCTCTTTCATCTGGAACAGCTCATGCTGGAGCTTCAGTAGACTTAGCTATTTTTTCTTTACATTTAGCAGGAATTTCATCAATTTTAGGTGCAGTAAATTTTATTACAACAGTAATTAATATACGATCTTCAGGAATTACTCTTGATCGAATACCTTTATTTGTTTGATCAGTAGTAATTACTGCAGTTTTATTACTTCTTTCTTTACCTGTTTTAGCTGGTGCTATTACTATGTTATTAACAGATCGAAATTTAAATACTTCATTCTTTGATCCAATTGGAGGAGGAGATCCAATTTTATATCAACATTTATTTTGATTTTTTGGTCACCCTGAAGTTTAAAGTTAA

> Sample 35

CTAGGGGTTGGGGGAAACTTCTTTAAGTTTACTAATTCGAGCAGAATTAAGTCAACCAGGTGTATTTATTGGAAATGATCAAATTTATAATGTTATTGTAACTGCTCATGCTTTTATTATAATTTTTTTTATAGTAATACCAATCATAATTGGAGGATTTGGAAATTGATTAGTTCCTTTAATGTTAGGAGCTCCAGATATGGCCTTTCCTCGAATAAATAATATAAGTTTTTGAATACTACCTCCTTCATTGACACTACTACTTTCAAGTAGTTTAGTAGAAAATGGAGCTGGGACTGGATGAACAGTGTATCCCCCTCTTTCATCTGGAACAGCTCATGCTGGAGCTTCAGTAGACTTAGCTATTTTTTCTTTACATTTAGCAGGAATTTCATCAATTTTAGGTGCAGTAAATTTTATTACAACAGTAATTAATATACGATCTTCAGGAATTACTCTTGATCGAATACCTTTATTTGTTTGATCAGTAGTAATTACTGCAGTTTTATTACTTCTTTCTTTACCTGTTTTAGCTGGTGCTATTACTATGTTATTAACAGATCGAAATTTAAATACTTCATTCTTTGATCCAATTGGAGGAGGAGATCCAATTTTATATCAACATTTATTTTGATTTTTTGGTCACCCTGAAGTTTAA

> Sample 36

CCTAATATTTTTTTATTTTTGGGGCTTGAGCTGGATAGTTGGAACTTCTTTAAGTTTACTAATTCGAGCAGAATTAAGTCAACCAGGTGTATTTATTGGAAATGATCAAATTTATAATGTTATTGTAACTGCTCATGCTTTTATTATAATTTTTTTTATAGTAATACCAATCATAATTGGAGGATTTGGAAATTGATTAGTTCCTTTAATGTTAGGAGCTCCAGATATGGCCTTTCCTCGAATAAATAATATAAGTTTTTGAATACTACCTCCTTCATTGACACTACTACTTTCAAGTAGTTTAGTAGAAAATGGAGCTGGGACTGGATGAACAGTGTATCCCCCTCTTTCATCTGGAACAGCTCATGCTGGAGCTTCAGTAGACTTAGCTATTTTTTCTTTACATTTAGCAGGAATTTCATCAATTTTAGGTGCAGTAAATTTTATTACAACAGTAATTAATATACGATCTTCAGGAATTACTCTTGATCGAATACCTTTATTTGTTTGATCAGTAGTAATTACTGCAGTTTTATTACTTCTTTCTTTACCTGTTTTAGCTGGTGCTATTACTATGTTATTAACAGATCGAAATTTAAATACTTCATTCTTTGATCCAATTGGAGGAGGAGATCCAATTTTATATCAACATTTATTTTGATTTTTTGGTCACCCTGAAGTTTAA

> Sample 37

CGTTGGGGTTTGGAGCTGGGATAGTTGGAACTTCTTTAAGTTTACTAATTCGAGCAGAATTAAGTCAACCAGGTGTATTTATTGGAAATGATCAAATTTATAATGTTATTGTAACTGCTCATGCTTTTATTATAATTTTTTTTATAGTAATACCAATCATAATTGGAGGATTTGGAAATTGATTAGTTCCTTTAATGTTAGGAGCTCCAGATATGGCCTTTCCTCGAATAAATAATATAAGTTTTTGAATACTACCTCCTTCATTGACACTACTACTTTCAAGTAGTTTAGTAGAAAATGGAGCTGGGACTGGATGAACAGTGTATCCCCCTCTTTCATCTGGAACAGCTCATGCTGGAGCTTCAGTAGACTTAGCTATTTTTTCTTTACATTTAGCAGGAATTTCATCAATTTTAGGTGCAGTAAATTTTATTACAACAGTAATTAATATACGATCTTCAGGAATTACTCTTGATCGAATACCTTTATTTGTTTGATCAGTAGTAATTACTGCAGTTTTATTACTTCTTTCTTTACCTGTTTTAGCTGGTGCTATTACTATGTTATTAACAGATCGAAATTTAAATACTTCATTTCTTTGATCCAATTGGAGGAGGAGATCCAATTTTATATCAACATTTATTTTTGATTTTTTGGTCACCCTGAAAGTTTAA

> Sample 38

CATCTTTATTTTTTGGGGGCTTGAGCTGGATAGTTGGAACTTCTTTAAGTTTACTAATTCGAGCAGAATTAAGTCAACCAGGTGTATTTATTGGAAATGATCAAATTTATAATGTTATTGTAACTGCTCATGCTTTTATTATAATTTTTTTTATAGTAATACCAATCATAATTGGAGGATTTGGAAATTGATTAGTTCCTTTAATGTTAGGAGCTCCAGATATGGCCTTTCCTCGAATAAATAATATAAGTTTTTGAATACTACCTCCTTCATTGACACTACTACTTTCAAGCAGTTTAGTAGAAAATGGAGCTGGGACTGGATGAACAGTGTATCCCCCTCTTTCATCTGGAACAGCTCATGCTGGAGCTTCAGTAGACTTAGCTATTTTTTCTTTACATTTAGCAGGAATTTCATCAATTTTAGGTGCAGTAAATTTTATTACAACAGTAATTAATATACGATCTTCAGGAATTACTCTTGATCGAATACCTTTATTTGTTTGATCAGTAGTAATTACTGCAGTTTTATTACTTCTTTCTTTACCTGTTTTAGCTGGTGCTATTACTATGTTATTAACAGATCGAAATTTAAATACTTCATTCTTTGATCCAATTGGAGGAGGAGATCCAATTTTATATCAACATTTATTTTGATTTTTTGGTCACCCTGAAGTTTA

> Sample 39

TGGGTTTGGGAACTTCTTTAAGTTTACTAATTCGAGCAGAATTAAGTCAACCAGGTGTATTTATTGGAAATGATCAAATTTATAATGTTATTGTAACTGCTCATGCTTTTATTATAATTTTTTTTATAGTAATACCAATCATAATTGGAGGATTTGGAAATTGATTAGTTCCTTTAATGTTAGGAGCTCCAGATATGGCCTTTCCTCGAATAAATAATATAAGTTTTTGAATACTACCTCCTTCATTGACACTACTACTTTCAAGTAGTTTAGTAGAAAATGGAGCTGGGACTGGATGAACAGTGTATCCCCCTCTTTCATCTGGAACAGCTCATGCTGGAGCTTCAGTAGACTTAGCTATTTTTTCTTTACATTTAGCAGGAATTTCATCAATTTTAGGTGCAGTAAATTTTATTACAACAGTAATTAATATACGATCTTCAGGAATTACTCTTGATCGAATACCTTTATTTGTTTGATCAGTAGTAATTACTGCAGTTTTATTACTTCTTTCTTTACCTGTTTTAGCTGGTGCTATTACTATGTTATTAACAGATCGAAATTTAAATACTTCATTCTTTGATCCAATTGGAGGAGGAGATCCAATTTTATATCAACATTTATTTTGATTTTTTGGTCACCCTGAAGTTTTAA

> Sample 40

GGAGCTGGGGAATAGTTGGAACTTCTTTAAGTTTACTAATTCGAGCAGAATTAAGTCAACCAGGTGTATTTATTGGAAATGATCAAATTTATAATGTTATTGTAACTGCTCATGCTTTTATTATAATTTTTTTTATAGTAATACCAATCATAATTGGAGGATTTGGAAATTGATTAGTTCCTTTAATGTTAGGAGCTCCAGATATGGCCTTTCCTCGAATAAATAATATAAGTTTTTGAATACTACCTCCTTCATTGACACTACTACTTTCAAGTAGTTTAGTAGAAAATGGAGCTGGGACTGGATGAACAGTGTATCCCCCTCTTTCATCTGGAACAGCTCATGCTGGAGCTTCAGTAGACTTAGCTATTTTTTCTTTACATTTAGCAGGAATTTCATCAATTTTAGGTGCAGTAAATTTTATTACAACAGTAATTAATATACGATCTTCAGGAATTACTCTTGATCGAATACCTTTATTTGTTTGATCAGTAGTAATTACTGCAGTTTTATTACTTCTTTCTTTACCTGTTTTAGCTGGTGCTATTACTATGTTATTAACAGATCGAAATTTAAATACTTCATTCTTTGATCCAATTGGAGGAGGAGATCCAATTTTATATCAACATTTTATTTTGATTTTTTGGTCACCCTGAAGTTTAAA

> Sample 41

CAAAGGGTTTGTGGGGAAACTTTTCTTTTAAAGGTTTACTAATTCGAGCAGAATTAAGTCAACCAGGTGTATTTATTGGAAATGATCAAATTTATAATGTTATTGTAACTGCTCATGCTTTTATTATAATTTTTTTTATAGTAATACCAATCATAATTGGAGGATTTGGAAATTGATTAGTTCCTTTAATGTTAGGAGCTCCAGATATGGCCTTTCCTCGAATAAATAATATAAGTTTTTGAATACTACCTCCTTCATTGACACTACTACTTTCAAGTAGTTTAGTAGAAAATGGAGCTGGGACTGGATGAACAGTGTATCCCCCTCTTTCATCTGGAACAGCTCATGCTGGAGCTTCAGTAGACTTAGCTATTTTTTCTTTACATTTAGCAGGAATTTCATCAATTTTAGGTGCAGTAAATTTTATTACAACAGTAATTAATATACGATCTTCAGGAATTACTCTTGATCGAATACCTTTATTTGTTTGATCAGTAGTAATTACTGCAGTTTTATTACTTCTTTCTTTACCTGTTTTAGCTGGTGCTATTACTATGTTATTAACAGATCGAAATTTAAATACTTCATTCTTTGATCCAATTGGAGGAGGAGATCCAATTTTATATCAACATTTATTTTGATTTTTTGGTCACCCTGAAGTTTAA

> Sample 42

TTGGGGGAATAGTTTGGAACTTCTTTAAGTTTACTAATTCGAGCAGAATTAAGTCAACCAGGTGTATTTATTGGAAATGATCAAATTTATAATGTTATTGTAACTGCTCATGCTTTTATTATAATTTTTTTTATAGTAATACCAATCATAATTGGAGGATTTGGAAATTGATTAGTTCCTTTAATGTTAGGAGCTCCAGATATGGCCTTTCCTCGAATAAATAATATAAGTTTTTGAATACTACCTCCTTCATTGACACTACTACTTTCAAGTAGTTTAGTAGAAAATGGAGCTGGGACTGGATGAACAGTGTATCCCCCTCTTTCATCTGGAACAGCTCATGCTGGAGCTTCAGTAGACTTAGCTATTTTTTCTTTACATTTAGCAGGAATTTCATCAATTTTAGGTGCAGTAAATTTTATTACAACAGTAATTAATATACGATCTTCAGGAATTACTCTTGATCGAATACCTTTATTTGTTTGATCAGTAGTAATTACTGCAGTTTTATTACTTCTTTCTTTACCTGTTTTAGCTGGTGCTATTACTATGTTATTAACAGATCGAAATTTAAATACTTCATTCTTTGATCCAATTGGAGGAGGAGATCCAATTTTATATCAACATTTATTTTGATTTTTTGGTCACCCTGAAGTTTAA

> Sample 43

TCGAAAGGCCAAGGAATTTTAACTTTCAACCATGGTGTTATTTATTGGAAATGATCAAATTTATAATGTTATTGTAACTGCTCATGCTTTTATTATAATTTTTTTTATAGTAATACCAATCATAATTGGAGGATTTGGAAATTGATTAGTTCCTTTAATGTTAGGAGCTCCAGATATGGCCTTTCCTCGAATAAATAATATAAGTTTTTGAATACTACCTCCTTCATTGACACTACTACTTTCAAGTAGTTTAGTAGAAAATGGAGCTGGGACTGGATGAACAGTGTATCCCCCTCTTTCATCTGGAACAGCTCATGCTGGAGCTTCAGTAGACTTAGCTATTTTTTCTTTACATTTAGCAGGAATTTCATCAATTTTAGGTGCAGTAAATTTTATTACAACAGTAATTAATATACTATCTTCAGGAATTACTCTTGATCGAATACCTTTATTTGTTTGATCAGTAGAAATTTACTGCAGTTTTATTACTTCTTTCTTTACCTGTTTTAGCTGGTGCTATAACTATGTTATTAACAGATCGAAATTTCAATACTTCATTCTTTGATCCAATTGGAGGAGGAGATCCAATTTTTATATCA

> Sample 44

TTCCTTTTTTAAAGTTTTTACTAATTCGAGCAGAATTAAGTCAACCAGGTGTATTTATTGGAAATGATCAAATTTATAATGTTATTGTAACTGCTCATGCTTTTATTATAATTTTTTTTATAGTAATACCAATCATAATTGGAGGATTTGGAAATTGATTAGTTCCTTTAATGTTAGGAGCTCCAGATATGGCCTTTCCTCGAATAAATAATATAAGTTTTTGAATACTACCTCCTTCATTGACACTACTACTTTCAAGTAGTTTAGTAGAAAATGGGGCTGGGACTGGATGAACAGTGTATCCCCCTCTTTCATCTGGAACAGCTCATGCTGGAGCTTCAGTAGACTTAGCTATTTTTTCTTTACATTTAGCAGGAATTTCATCAATTTTAGGTGCAGTAAATTTTATTACAACAGTAATTAATATACGATCTTCAGGAATTACTCTTGATCGAATACCTTTATTTGTTTGATCAGTAGTAATTACTGCAGTTTTATTACTTCTTTCTTTACCTGTTTTAGCTGGTGCTATTACTATGTTATTAACAGATCGAAATTTAAATACTTCATTCTTTGATCCAATTGGAGGAGGAGATCCAATTTTATATCAACATTTATTTTGATTTTTTGGTCACCCTGAAGTTTTAA

> Sample 45

ATGCTCGATGGTTGCTCTGGATAGTTGGAACTTCTTTAAGTTTACTAATTCGAGCAGAATTAAGTCGACCAGGTGTATTTATTGGAAATGATCAAATTTATAATGTTATTGTAACTGCTCATGCTTTTATTATAATTTTTTTTATAGTAATACCAATCATAATTGGAGGATTTGGAAATTGATTAGTTCCTTTAATGTTAGGAGCTCCAGATATGGCCTTTCCTCGAATAAATAATATAAGTTTTTGAATACTACCTCCTTCATTGACACTACTACTTTCAAGTAGTTTAGTAGAAAATGGAGCTGGGACTGGATGAACAGTGTATCCCCCTCTTTCATCTGGAACAGCTCATGCTGGAGCTTCAGTAGACTTAGCTATTTTTTCTTTACATTTAGCAGGAATTTCATCAATTTTAGGTGCAGTAAATTTTATTACAACAGTAATTAATATACGATCTTCAGGAATTACTCTTGATCGAATACCTTTATTTGTTTGATCAGTAGTAATTACTGCAGTTTTATTACTTCTTTCTTTACCTGTTTTAGCTGGTGCTATTACTATGTTATTAACAGATCGAAATTTAAATACTTCATTCTTTGATCCAATTGGAGGAGGAGATCCAATTTTATATCAACATTTATTTTGATTTTTTGGTCACCCTGAAGTTTAA

> Sample 46

CGGGGGGCTTGGAGCTGGGAATAGTTGGAACTTCTTTAAGTTTACTAATTCGAGCAGAATTAAGTCAACCAGGTGTATTTATTGGAAATGATCAAATTTATAATGTTATTGTAACTGCTCATGCTTTTATTATAATTTTTTTTATAGTAATACCAATCATAATTGGAGGATTTGGAAATTGATTAGTTCCTTTAATGTTAGGAGCTCCAGATATGGCCTTTCCTCGAATAAATAATATAAGTTTTTGAATACTACCTCCTTCATTGACACTACTACTTTCAAGTAGTTTAGTAGAAAATGGAGCTGGGACTGGATGAACAGTGTATCCCCCTCTTTCATCTGGAACAGCTCATGCTGGAGCTTCAGTAGACTTAGCTATTTTTTCTTTACATTTAGCAGGAATTTCATCAATTTTAGGTGCAGTAAATTTTATTACAACAGTAATTAATATACGATCTTCAGGAATTACTCTTGATCGAATACCTTTATTTGTTTGATCAGTAGTAATTACTGCAGTTTTATTACTTCTTTCTTTACCTGTTTTAGCTGGTGCTATTACTATGTTATTAACAGATCGAAATTTAAATACTTCATTCTTTGATCCAATTGGAGGAGGAGATCCAATTTTATATCAACATTTATTTTGATTTTTTGGTCACCCTGAAGTTTAA

> Sample 47

TGGGTTTTGGGGGAACTTCTTTAAGTTTACTAATTCGAGCAGAATTAAGTCAACCAGGTGTATTTATTGGAAATGATCAAATTTATAATGTTATTGTAACTGCTCATGCTTTTATTATAATTTTTTTTATAGTAATACCAATCATAATTGGAGGATTTGGAAATTGATTAGTTCCTTTAATGTTAGGAGCTCCAGATATGGCCTTTCCTCGAATAAATAATATAAGTTTTTGAATACTACCTCCTTCATTGACACTACTACTTTCAAGTAGTTTAGTAGAAAATGGAGCTGGGACTGGATGAACAGTGTATCCCCCTCTTTCATCTGGAACAGCTCATGCTGGAGCTTCAGTAGACTTAGCTATTTTTTCTTTACATTTAGCAGGAATTTCATCAATTTTAGGTGCAGTAAATTTTATTACAACAGTAATTAATATACGATCTTCAGGAATTACTCTTGATCGAATACCTTTATTTGTTTGATCAGTAGTAATTACTGCAGTTTTATTACTTCTTTCTTTACCTGTTTTAGCTGGTGCTATTACTATGTTATTAACAGATCGAAATTTAAATACTTCATTCTTTGATCCAATTGGAGGAGGAGATCCAATTTTATATCAACATTTATTTTGATTTTTTGGTCACCCTGAAGTTTAA

> Sample 48

TGGGGGGGCTTGGGAGCTGGGGAATAGTTGGAACTTCTTTAAGTTTACTAATTCGAGCAGAATTAAGTCAACCAGGTGTATTTATTGGAAATGATCAAATTTATAATGTTATTGTAACTGCTCATGCTTTTATTATAATTTTTTTTATAGTAATACCAATCATAATTGGAGGATTTGGAAATTGATTAGTTCCTTTAATGTTAGGAGCTCCAGATATGGCCTTTCCTCGAATAAATAATATAAGTTTTTGAATACTACCTCCTTCATTGACACTACTACTTTCAAGTAGTTTAGTAGAAAATGGAGCTGGGACTGGATGAACAGTGTATCCCCCTCTTTCATCTGGAACAGCTCATGCTGGAGCTTCAGTAGACTTAGCTATTTTTTCTTTACATTTAGCAGGAATTTCATCAATTTTAGGTGCAGTAAATTTTATTACAACAGTAATTAATATACGATCTTCAGGAATTACTCTTGATCGAATACCTTTATTTGTTTGATCAGTAGTAATTACTGCAGTTTTATTACTTCTTTCTTTACCTGTTTTAGCTGGTGCTATTACTATGTTATTAACAGATCGAAATTTAAATACTTCATTCTTTGATCCAATTGGAGGAGGAGATCCAATTTTATATCAACATTTATTTTGATTTTTTGGTCACCCTGAAGTTTAA

> Sample 49

AAAATTAATTTTTTCCGGGCTTGAGCTGGATAGTTGGAACTTCTTTAAGTTTACTAATTCGAGCAGAATTAAGTCAACCAGGTGTATTTATTGGAAATGATCAAATTTATAATGTTATTGTAACTGCTCATGCTTTTATTATAATTTTTTTTATAGTAATACCAATCATAATTGGAGGATTTGGAAATTGATTAGTTCCTTTAATGTTAGGAGCTCCAGATATGGCCTTTCCTCGAATAAATAATATAAGTTTTTGAATACTACCTCCTTCATTGACACTACTACTTTCAAGTAGTTTAGTAGAAAATGGAGCTGGGACTGGATGAACAGTGTATCCCCCTCTTTCATCTGGAACAGCTCATGCTGGAGCTTCAGTAGACTTAGCTATTTTTTCTTTACATTTAGCAGGAATTTCATCAATTTTAGGTGCAGTAAATTTTATTACAACAGTAATTAATATACGATCTTCAGGAATTACTCTTGATCGAATACCTTTATTTGTTTGATCAGTAGTAATTACTGCAGTTTTATTACTTCTTTCTTTACCTGTTTTAGCTGGTGCTATTACTATGTTATTAACAGATCGAAATTTAAATACTTCATTCTTTGATCCAATTGGAGGAGGAGATCCAATTTTATATCAACATTTATTTTGATTTTTTGGTCACCCTGAAGTTTA

> Sample 50

AAGCCTGCTTGAGCTGGAATAGTTGGAACTTCTTTAAGTTTACTAATTCGAGCAGAATTAAGTCAACCAGGTGTATTTATTGGAAATGATCAAATTTATAATGTTATTGTAACTGCTCATGCTTTTATTATAATTTTTTTTATAGTAATACCAATCATAATTGGAGGATTTGGAAATTGATTAGTTCCTTTAATGTTAGGAGCTCCAGATATGGCCTTTCCTCGAATAAATAATATAAGTTTTTGAATACTACCTCCTTCATTGACACTACTACTTTCAAGTAGTTTAGTAGAAAATGGAGCTGGGACTGGATGAACAGTGTATCCCCCTCTTTCATCTGGAACAGCTCATGCTGGAGCTTCAGTAGACTTAGCTATTTTTTCTTTACATTTAGCAGGAATTTCATCAATTTTAGGTGCAGTAAATTTTATTACAACAGTAATTAATATACGATCTTCAGGAATTACTCTTGATCGAATACCTTTATTTGTTTGATCAGTAGTAATTACTGCAGTTTTATTACTTCTTTCTTTACCTGTTTTAGCTGGTGCTATTACTATGTTATTAACAGATCGAAATTTAAATACTTCATTCTTTGATCCAATTGGAGGAGGAGATCCAATTTTATATCAACATTTATTTTGATTTTTTGGTCACCCTGGAAGTTTAA

> Sample 51

AGGTTTGGGGGAACCTTTCCTTTAAGTTTACTAATTCGAGCAGAATTAAGTCAACCAGGTGTATTTATTGGAAATGATCAAATTTATAATGTTATTGTAACTGCTCATGCTTTTATTATAATTTTTTTTATAGTAATACCAATCATAATTGGAGGATTTGGAAATTGATTAGTTCCTTTAATGTTAGGAGCTCCAGATATGGCCTTTCCTCGAATAAATAATATAAGTTTTTGAATACTACCTCCTTCATTGACACTACTACTTTCAAGTAGTTTAGTAGAAAATGGAGCTGGGACTGGATGAACAGTGTATCCCCCTCTTTCATCTGGAACAGCTCATGCTGGAGCTTCAGTAGACTTAGCTATTTTTTCTTTACATTTAGCAGGAATTTCATCAATTTTAGGTGCAGTAAATTTTATTACAACAGTAATTAATATACGATCTTCAGGAATTACTCTTGATCGAATACCTTTATTTGTTTGATCAGTAGTAATTACTGCAGTTTTATTACTTCTTTCTTTACCTGTTTTAGCTGGTGCTATTACTATGTTATTAACAGATCGAAATTTAAATACTTCATTCTTTGATCCAATTGGAGGAGGAGATCCAATTTTATATCAACATTTATTTTGATTTTTTGGTCACCCTGAAAGTTTAA

> Sample 52

CTTTTCCCTTTTTAAAGGGTTTTACTAATTCGGAGGCAGAATTAAGTCAACCAGGTGTATTTATTGGAAATGATCAAATTTATAATGTTATTGTAACTGCTCATGCTTTTATTATAATTTTTTTTATAGTAATACCAATCATAATTGGAGGATTTGGAAATTGATTAGTTCCTTTAATGTTAGGAGCTCCAGATATGGCCTTTCCTCGAATAAATAATATAAGTTTTTGAATACTACCTCCTTCATTGACACTACTACTTTCAAGTAGTTTAGTAGAAAATGGAGCTGGGACTGGATGAACAGTGTATCCCCCTCTTTCATCTGGAACAGCTCATGCTGGAGCTTCAGTAGACTTAGCTATTTTTTCTTTACATTTAGCAGGAATTTCATCAATTTTAGGTGCAGTAAATTTTATTACAACAGTAATTAATATACGATCTTCAGGAATTACTCTTGATCGAATACCTTTATTTGTTTGATCAGTAGTAATTACTGCAGTTTTATTACTTCTTTCTTTACCTGTTTTAGCTGGTGCTATTACTATGTTATTAACAGATCGAAATTTAAATACTTCATTCTTTGATCCAATTGGAGGAGGAGATCCAATTTTATATCAACATTTATTTTGATTTTTTGGTCACCCTGAAGTTTAA

> Sample 54

CCGAGCTGGGAGCCTGGGGAATAGTTGGAACTTCTTTAAGTTTACTAATTCGAGCAGAATTAAGTCAACCAGGTGTATTTATTGGAAATGATCAAATTTATAATGTTATTGTAACTGCTCATGCTTTTATTATAATTTTTTTTATAGTAATACCAATCATAATTGGAGGATTTGGAAATTGATTAGTTCCTTTAATGTTAGGAGCTCCAGATATGGCCTTTCCTCGAATAAATAATATAAGTTTTTGAATACTACCTCCTTCATTGACACTACTACTTTCAAGTAGTTTAGTAGAAAATGGAGCTGGGACTGGATGAACAGTGTATCCCCCTCTTTCATCTGGAACAGCTCATGCTGGAGCTTCAGTAGACTTAGCTATTTTTTCTTTACATTTAGCAGGAATTTCATCAATTTTAGGTGCAGTAAATTTTATTACAACAGTAATTAATATACGATCTTCAGGAATTACTCTTGATCGAATACCTTTATTTGTTTGATCAGTAGTAATTACTGCAGTTTTATTACTTCTTTCTTTACCTGTTTTAGCTGGTGCTATTACTATGTTATTAACAGATCGAAATTTAAATACTTCATTCTTTGATCCAATTGGAGGAGGAGATCCAATTTTATATCAACATTTATTTTGATTTTTTGGTCACCCTGAAGTTTAA

> Sample 55

CGGGGGCTTGGAGCTGGGGAATAGTTGGAACTTCTTTAAGTTTACTAATTCGAGCAGAATTAAGTCAACCAGGTGTATTTATTGGAAATGATCAAATTTATAATGTTATTGTAACTGCTCATGCTTTTATTATAATTTTTTTTATAGTAATACCAATCATAATTGGAGGATTTGGAAATTGATTAGTTCCTTTAATGTTAGGAGCTCCAGATATGGCCTTTCCTCGAATAAATAATATAAGTTTTTGAATACTACCTCCTTCATTGACACTACTACTTTCAAGTAGTTTAGTAGAAAATGGAGCTGGGACTGGATGAACAGTGTATCCCCCTCTTTCATCTGGAACAGCTCATGCTGGAGCTTCAGTAGACTTAGCTATTTTTTCTTTACATTTAGCAGGAATTTCATCAATTTTAGGTGCAGTAAATTTTATTACAACAGTAATTAATATACGATCTTCAGGAATTACTCTTGATCGAATACCTTTATTTGTTTGATCAGTAGTAATTACTGCAGTTTTATTACTTCTTTCTTTACCTGTTTTAGCTGGTGCTATTACTATGTTATTAACAGATCGAAATTTAAATACTTCATTCTTTGATCCAATTGGAGGAGGAGATCCAATTTTATATCAACATTTATTTTGATTTTTTGGTCACCCTGAAGTTTAA

> Sample 56

AGGGGGGGGCTTTGAGGCTGGGAATAGTTGGAACTTCTTTAAGTTTACTAATTCGAGCAGAATTAAGTCAACCAGGTGTATTTATTGGAAATGATCAAATTTATAATGTTATTGTAACTGCTCATGCTTTTATTATAATTTTTTTTATAGTAATACCAATCATAATTGGAGGATTTGGAAATTGATTAGTTCCTTTAATGTTAGGAGCTCCAGATATGGCCTTTCCTCGAATAAATAATATAAGTTTTTGAATACTACCTCCTTCATTGACACTACTACTTTCAAGTAGTTTAGTAGAAAATGGAGCTGGGACTGGATGAACAGTGTATCCCCCTCTTTCATCTGGAACAGCTCATGCTGGAGCTTCAGTAGACTTAGCTATTTTTTCTTTACATTTAGCAGGAATTTCATCAATTTTAGGTGCAGTAAATTTTATTACAACAGTAATTAATATACGATCTTCAGGAATTACTCTTGATCGAATACCTTTATTTGTTTGATCAGTAGTAATTACTGCAGTTTTATTACTTCTTTCTTTACCTGTTTTAGCTGGTGCTATTACTATGTTATTAACAGATCGAAATTTAAATACTTCATTCTTTGATCCAATTGGAGGAGGAGATCCAATTTTATATCAACATTTATTTTGATTTTTTGGTCACCCTGAAGTTTAA

> Sample 57

AGGAGGGCTGGGGAATAAGTTGGGGAACTTCCTTTAAGTTTACTAATTCCAGCAGAATTAAGTCAACCAGGTGTATTTATTGGAAATGATCAAATTTATAATGTTATTGTAACTGCTCATGCTTTTATTATAATTTTTTTTATAGTAATACCAATCATAATTGGAGGATTTGGAAATTGATTAGTTCCTTTAATGTTAGGAGCTCCAGATATGGCCTTTCCTCGAATAAATAATATAAGTTTTTGAATACTACCTCCTTCATTGACACTACTACTTTCAAGTAGTTTAGTAAAAAATGGAGCTGGGACTGGATGAACAGCGTATCCCCCTCTTTCATCTGGAACAGCTCATGCTGGAGCTTCAGTACACTTAGCTATTTTTTCTTTACATTTAGCAGGAATTTCATCAATTTTAGGTGCAGTAAATTTTATTACAACAGTAATTAATATACGATCTTCAGGAATTACTCTTGATCGAATACCTTTATTTGTTTGATCAGTAGTAATTACTGCAGTTTTATTACTTCTTTCTTTACCTGTTTTAGCTGGTGCTATTACTATGTTATTAACAGATCGAAATTTAAATACTTCATTCTTTG

> Sample 58

TGGGGATTAGGTTTGGAACTTCTTTAAGTTTACTAATTCGAGCAGAATTAAGTCAACCAGGTGTATTTATTGGAAATGATCAAATTTATAATGTTATTGTAACTGCTCATGCTTTTATTATAATTTTTTTTATAGTAATACCAATCATAATTGGAGGATTTGGAAATTGATTAGTTCCTTTAATGTTAGGAGCTCCAGATATGGCCTTTCCTCGAATAAATAATATAAGTTTTTGAATACTACCTCCTTCATTGACACTACTACTTTCAAGTAGTTTAGTAGAAAATGGAGCTGGGACTGGATGAACAGTGTATCCCCCTCTTTCATCTGGAACAGCTCATGCTGGAGCTTCAGTAGACTTAGCTATTTTTTCTTTACATTTAGCAGGAATTTCATCAATTTTAGGTGCAGTAAATTTTATTACAACAGTAATTAATATACGATCTTCAGGAATTACTCTTGATCGAATACCTTTATTTGTTTGATCAGTAGTAATTACTGCAGTTTTATTACTTCTTTCTTTACCTGTTTTAGCTGGTGCTATTACTATGTTATTAACAGATCGAAATTTAAATACTTCATTCTTTGATCCAATTGGAGGAGGAGATCCAATTTTATATCAACATTTATTTTGATTTTTTGGTCACCCTGAAGTTTAAA

> Sample 59

CAGGTTTGGGGGAAACTTCCTTTTTAAGTTTTACTAATTTCGAGCAGAATTTAAGTCAACCAGGTTGTATTTATTGGAAATGATCAAATTTATAATGTTATTGTAACTGCTCATGCTTTTATTATAATTTTTTTTATAGTAATACCAATCATAATTGGAGGATTTGGAAATTGATTAGTTCCTTTAATGTTAGGAGCTCCAGATATGGCCTTTCCTCGAATAAATAATATAAGTTTTTGAATACTACCTCCTTCATTGACACTACTACTTTCAAGTAGTTTAGTAGAAAATGGGGCTGGGACTGGATGAACAGTGTATCCCCCTCTTTCATCTGGAACAGCTCATGCTGGAGCTTCAGTAGACTTAGCTATTTTTTCTTTACATTTAGCAGGAATTTCATCAATTTTAGGTGCAGTAAATTTTATTACAACAGTAATTAATATACGATCTTCAGGAATTACTCTTGATCGAATACCTTTATTTGTTTGATCAGTAGTAATTACTGCAGTTTTTATTACTTCTTTCTTTACCTGTTTTAGCTGGTGCTATTACTATGTTATTAACAGATCGAAATTTAAATACTTCATTCTTTGATCCAATTGGAGGAGGAGATCCAATTTTATATCAACATTTATTTTGATTTTTTTGGTCACCCTGAAGTTTAA

> Sample 60

AAATTTTTATTTTTGCGGGCTTGAGCTGGAATAGTTGGAACTTCTTTAAGTTTACTAATTCGAGCAGAATTAAGTCAACCAGGTGTATTTATTGGAAATGATCAAATTTATAATGTTATTGTAACTGCTCATGCTTTTATTATAATTTTTTTTATAGTAATACCAATCATAATTGGAGGATTTGGAAATTGATTAGTTCCTTTAATGTTAGGAGCTCCAGATATGGCCTTTCCTCGAATAAATAATATAAGTTTTTGAATACTACCTCCTTCATTGACACTACTACTTTCAAGTAGTTTAGTAGAAAATGGGGCTGGGACTGGATGAACAGTGTATCCCCCTCTTTCATCTGGAACAGCTCATGCTGGAGCTTCAGTAGACTTAGCTATTTTTTCTTTACATTTAGCAGGAATTTCATCAATTTTAGGTGCAGTAAATTTTATTACAACAGTAATTAATATACGATCTTCAGGAATTACTCTTGATCGAATACCTTTATTTGTTTGATCAGTAGTAATTACTGCAGTTTTATTACTTCTTTCTTTACCTGTTTTAGCTGGTGCTATTACTATGTTATTAACAGATCGAAATTTAAATACTTCATTCTTTGATCCAATTGGAGGAGGAGATCCAATTTTATATCAACATTTATTTTGATTTTTTGGTCACCCTGAAGTTTAA

> Sample 61

TTGCGGGGGCTTGAGCCTGGAATAGCTGGAACTTCTTTAAGTTTACTAATTCGAGCAGAATTAAGTCAACCAGGTGTATTTATTGGAAATGATCAAATTTATAATGTTATTGTAACTGCTCATGCTTTTATTATAATTTTTTTTATAGTAATACCAATCATAATTGGAGGATTTGGAAATTGATTAGTTCCTTTAATGTTAGGAGCTCCAGATATGGCCTTTCCTCGAATAAATAATATAAGTTTTTGAATACTACCTCCTTCATTGACACTACTACTTTCAAGTAGTTTAGTAGAAAATGGAGCTGGGACTGGATGAACAGTGTATCCCCCTCTTTCATCTGGAACAGCTCATGCTGGAGCTTCAGTAGACTTAGCTATTTTTTCTTTACATTTAGCAGGAATTTCATCAATTTTAGGTGCAGTAAATTTTATTACAACAGTAATTAATATACGATCTTCAGGAATTACTCTTGATCGAATACCTTTATTTGTTTGATCAGTAGTAATTACTGCAGTTTTATTACTTCTTTCTTTACCTGTTTTAGCTGGTGCTATTACTATGTTATTAACAGATCGAAATTTAAATACTTCATTTCTTTGATCCAATTGGAGGAGGAGATCCAATTTTATATCAACATTTATTTTGATTTTTTGGTCACCCTGAAGTTTAA

> Sample 62

CAAAATATTTTATTTTTTGGGGCTTGAGCTGGATAGTTGGAACTTCTTTAAGTTTACTAATTCGAGCAGAATTAAGTCAACCAGGTGTATTTATTGGAAATGATCAAATTTATAATGTTATTGTAACTGCTCATGCTTTTATTATAATTTTTTTTATAGTAATACCAATCATAATTGGAGGATTTGGAAATTGATTAGTTCCTTTAATGTTAGGAGCTCCAGATATGGCCTTTCCTCGAATAAATAATATAAGTTTTTGAATACTACCTCCTTCATTGACACTACTACTTTCAAGTAGTTTAGTAGAAAATGGAGCTGGGACTGGATGAACAGTGTATCCCCCTCTTTCATCTGGAACAGCTCATGCTGGAGCTTCAGTAGACTTAGCTATTTTTTCTTTACATTTAGCAGGAATTTCATCAATTTTAGGTGCAGTAAATTTTATTACAACAGTAATTAATATACGATCTTCAGGAATTACTCTTGATCGAATACCTTTATTTGTTTGATCAGTAGTAATTACTGCAGTTTTATTACTTCTTTCTTTACCTGTTTTAGCTGGTGCTATTACTATGTTATTAACAGATCGAAATTTAAATACTTCATTCTTTGATCCAATTGGAGGAGGAGATCCAATTTTATATCAACATTTATTTTGATTTTTTGGTCACCCTGAAGTTTAA

> Sample 63

CGGGGGGGGCTTGGAGCCTGGAATAGTTGGAACTTCTTTAAGTTTACTAATTCGAGCAGAATTAAGTCAACCAGGTGTATTTATTGGAAATGATCAAATTTATAATGTTATTGTAACTGCTCATGCTTTTATTATAATTTTTTTTATAGTAATACCAATCATAATTGGAGGATTTGGAAATTGATTAGTTCCTTTAATGTTAGGAGCTCCAGATATGGCCTTTCCTCGAATAAATAATATAAGTTTTTGAATACTACCTCCTTCATTGACACTACTACTTTCAAGTAGTTTAGTAGAAAATGGAGCTGGGACTGGATGAACAGTGTATCCCCCTCTTTCATCTGGAACAGCTCATGCTGGAGCTTCAGTAGACTTAGCTATTTTTTCTTTACATTTAGCAGGAATTTCATCAATTTTAGGTGCAGTAAATTTTATTACAACAGTAATTAATATACGATCTTCAGGAATTACTCTTGATCGAATACCTTTATTTGTTTGATCAGTAGTAATTACTGCAGTTTTATTACTTCTTTCTTTACCTGTTTTAGCTGGTGCTATTACTATGTTATTAACAGATCGAAATTTAAATACTTCATTCTTTGATCCAATTGGAGGAGGAGATCCAATTTTATATCAACATTTATTTTGATTTTTTGGTCACCCTGAAGTTTAA

> Sample 64

TTATTTTTTTTTGGGGGCTTGAGCTGGAATAGTTGGAACTTCTTTAAGGTTTACTAATTCGAGCAGGAATTAAGTCAACCAGGTGTATTTATTGGAAATGATCAAATTTATAATGTTATTGTAACTGCTCATGCTTTTATTATAATTTTTTTTATAGTAATACCAATCATAATTGGAGGATTTGGAAATTGATTAGTTCCTTTAATGTTAGGAGCTCCAGATATGGCCTTTCCTCGAATAAATAATATAAGTTTTTGAATACTACCTCCTTCATTGACACTACTACTTTCAAGTAGTTTAGTAGAAAATGGAGCTGGGACTGGATGAACAGTGTATCCCCCTCTTTCATCTGGAACAGCTCATGCTGGAGCTTCAGTAGACTTAGCTATTTTTTCTTTACATTTAGCAGGAATTTCATCAATTTTAGGTGCAGTAAATTTTATTACAACAGTAATTAATATACGATCTTCAGGAATTACTCTTGATCGAATACCTTTATTTGTTTGATCAGTAGTAATTACTGCAGTTTTATTACTTCTTTCTTTACCTGTTTTAGCTGGTGCTATTACTATGTTATTAACAGATCGAAATTTAAATACTTCATTCTTTGATCCAATTGGAGGAGGAGATCCAATTTTATATCAACATTTATTTTGATTTTTTGGTCACCCTGAAGTTTAA

> Sample 65

AATTTTTTGGGGGGGCTTGAGCCTGGAATAGTTGGAACTTCTTTAAGTTTACTAATTCGAGCAGAATTAAGTCAACCAGGTGTATTTATTGGAAATGATCAAATTTATAATGTTATTGTAACTGCTCATGCTTTTATTATAATTTTTTTTATAGTAATACCAATCATAATTGGAGGATTTGGAAATTGATTAGTTCCTTTAATGTTAGGAGCTCCAGATATGGCCTTTCCTCGAATAAATAATATAAGTTTTTGAATACTACCTCCTTCATTGACACTACTACTTTCAAGTAGTTTAGTAGAAAATGGAGCTGGGACTGGATGAACAGTGTATCCCCCTCTTTCATCTGGAACAGCTCATGCTGGAGCTTCAGTAGACTTAGCTATTTTTTCTTTACATTTAGCAGGAATTTCATCAATTTTAGGTGCAGTAAATTTTATTACAACAGTAATTAATATACGATCTTCAGGAATTACTCTTGATCGAATACCTTTATTTGTTTGATCAGTAGTAATTACTGCAGTTTTATTACTTCTTTCTTTACCTGTTTTAGCTGGTGCTATTACTATGTTATTAACAGATCGAAATTTAAATACTTCATTCTTTGATCCAATTGGAGGAGGAGATCCAATTTTATATCAACATTTATTTTGATTTTTTGGTCACCCTGAAGTTTAA

> Sample 66

TGGGGGGGGGGGTTTGGAGCCTGGGAATAGTTTGGAACTTCTTTAAGTTTACTAATTCGAGCAGAATTAAGTCAACCAGGTGTATTTATTGGAAATGATCAAATTTATAATGTTATTGTAACTGCTCATGCTTTTATTATAATTTTTTTTATAGTAATACCAATCATAATTGGAGGATTTGGAAATTGATTAGTTCCTTTAATGTTAGGAGCTCCAGATATGGCCTTTCCTCGAATAAATAATATAAGTTTTTGAATACTACCTCCTTCATTGACACTACTACTTTCAAGTAGTTTAGTAGAAAATGGAGCTGGGACTGGATGAACAGTGTATCCCCCTCTTTCATCTGGAACAGCTCATGCTGGAGCTTCAGTAGACTTAGCTATTTTTTCTTTACATTTAGCAGGAATTTCATCAATTTTAGGTGCAGTAAATTTTATTACAACAGTAATTAATATACGATCTTCAGGAATTACTCTTGATCGAATACCTTTATTTGTTTGATCAGAAGTAATTACTGCAGTTTTATTACTTCTTTCTTTACCTGTTTTAGCTGGTGCTATTACTATGTTATTAACAGATCGAAATTTAAATACTTCATTCTTTGATCCAATTGGAGGAGGAGATCCAATTTTATATCAACATTTATTTTTGATTTTTTTGGCACCCTGAAGTTTAAC

> Sample 67

CTGGGGGGCTTGAGCTGGAATAGTTGGAACTTCTTTAAGTTTACTAATTCGAGCAGAATTAAGTCAACCAGGTGTATTTATTGGAAATGATCAAATTTATAATGTTATTGTAACTGCTCATGCTTTTATTATAATTTTTTTTATAGTAATACCAATCATAATTGGAGGATTTGGAAATTGATTAGTTCCTTTAATGTTAGGAGCTCCAGATATGGCCTTTCCTCGAATAAATAATATAAGTTTTTGAATACTACCTCCTTCATTGACACTACTACTTTCAAGTAGTTTAGTAGAAAATGGAGCTGGGACTGGATGAACAGTGTATCCCCCTCTTTCATCTGGAACAGCTCATGCTGGAGCTTCAGTAGACTTAGCTATTTTTTCTTTACATTTAGCAGGAATTTCATCAATTTTAGGTGCAGTAAATTTTATTACAACAGTAATTAATATACGATCTTCAGGAATTACTCTTGATCGAATACCTTTATTTGTTTGATCAGTAGTAATTACTGCAGTTTTATTACTTCTTTCTTTACCTGTTTTAGCTGGTGCTATTACTATATTATTAACAGATCGAAATTTAAATACTTCATTCTTTGATCCAATTGGAGGAGGAGATCCAATTTTATATCAACATTTATTTTGATTTTTTGGTCACCCTGAAGTTTAA

> Sample 68

CGGGGGGCCTTTGAGCTGGAAATAGTTGGAACTTCTTTAAGTTTACTAATTCGAGCAGAATTAAGTCAACCAGGTGTATTTATTGGAAATGATCAAATTTATAATGTTATTGTAACTGCTCATGCTTTTATTATAATTTTTTTTATAGTAATACCAATCATAATTGGAGGATTTGGAAATTGATTAGTTCCTTTAATGTTAGGAGCTCCAGATATGGCCTTTCCTCGAATAAATAATATAAGTTTTTGAATACTACCTCCTTCATTGACACTACTACTTTCAAGTAGTTTAGTAGAAAATGGAGCTGGGACTGGATGAACAGTGTATCCCCCTCTTTCATCTGGAACAGCTCATGCTGGAGCTTCAGTAGACTTAGCTATTTTTTCTTTACATTTAGCAGGAATTTCATCAATTTTAGGTGCAGTAAATTTTATTACAACAGTAATTAATATACGATCTTCAGGAATTACTCTTGATCGAATACCTTTATTTGTTTGATCAGTAGTAATTACTGCAGTTTTATTACTTCTTTCTTTACCTGTTTTAGCTGGTGCTATTACTATGTTATTAACAGATCGAAATTTAAATACTTCATTCTTTGATCCAATTGGAGGAGGAGATCCAATTTTATATCAACATTTATTTTGATTTTTTGGTCACCCTGAAGTTTAA

> Sample 69

TGGGGGGCTTGAGCTGGAATAGTTGGAACTTCTTTAAGTTTACTAATTCGAGCAGAATTAAGTCAACCAGGTGTATTTATTGGAAATGATCAAATTTATAATGTTATTGTAACTGCTCATGCTTTTATTATAATTTTTTTTATAGTAATACCAATCATAATTGGAGGATTTGGAAATTGATTAGTTCCTTTAATGTTAGGAGCTCCAGATATGGCCTTTCCTCGAATAAATAATATAAGTTTTTGAATACTACCTCCTTCATTGACACTACTACTTTCAAGTAGTTTAGTAGAAAATGGAGCTGGGACTGGATGAACAGTGTATCCCCCTCTTTCATCTGGAACAGCTCATGCTGGAGCTTCAGTAGACTTAGCTATTTTTTCTTTACATTTAGCAGGAATTTCATCAATTTTAGGTGCAGTAAATTTTATTACAACAGTAATTAATATACGATCTTCAGGAATTACTCTTGATCGAATACCTTTATTTGTTTGATCAGTAGTAATTACTGCAGTTTTATTACTTCTTTCTTTACCTGTTTTAGCTGGTGCTATTACTATGTTATTAACAGATCGAAATTTAAATACTTCATTCTTTGATCCAATTGGAGGAGGAGATCCAATTTTATATCAACATTTATTTTGATTTTTTGGTCACCCTGGAAGTTTAA

> Sample 1.1

CTTTATTTTCGGTATTTGATCTGGAATAGTCGGAACTTCACTAAGAGTTTTAATTCGTATTGAACTTAGACATCCTGGTATATTTATTGGAAATGATCAAATTTATAATGTAATTGTTACTGCTCATGCTTTTATTATAATTTTTTTTATAGTAATACCTATCATAATTGGAGGATTTGGAAACTGACTAGTACCCTTAATACTAGGAGCCCCTGATATAGCTTTTCCTCGAATAAATAATATAAGTTTTTGAATATTACCCCCCTCTTTAACACTGCTGCTTTCTAGTTCTATAGTAGAAAACGGAGCTGGAACAGGGTGAACGGTTTATCCTCCTCTTTCTTCTGGAACAGCTCATGCTGGGGCTTCAGTTGATTTAGCAATTTTTTCTTTACATTTAGCGGGAATCTCATCTATTTTAGGAGCAGTAAATTTTATTACAACTGTAATTAATATACGATCAGCTGGTATTACTCTTGATCGACTACCTTTATTTGTGTGATCAGTAGTAATTACAGCTATTTTATTACTTCTTTCTCTACCCGTATTAGCCGGAGCTATTACTATATTATTAACAGACCGAAATTTAAATACATCTTTTTTTGATCCAATTGGAGGGGGAGACCCCTTATTT

> Sample 2.1

ACTTTATTTTCGGTATTTGATCTGGAATAGTCGGAACTTCACTAAGAGTTTTAATTCGTATTGAACTTAGACATCCTGGTATATTTATTGGAAATGATCAAATTTATAATGTAATTGTTACTGCTCATGCTTTTATTATAATTTTTTTTATAGTAATACCTATCATAATTGGAGGATTTGGAAACTGACTAGTACCCTTAATACTAGGAGCCCCTGATATAGCTTTTCCTCGAATAAATAATATAAGTTTTTGAATATTACCCCCCTCTTTAACACTGCTGCTTTCTAGTTCTATAGTAGAAAACGGAGCTGGAACAGGGTGAACGGTTTATCCTCCTCTTTCTTCTGGAACAGCTCATGCTGGGGCTTCAGTTGATTTAGCAATTTTTTCTTTACATTTAGCGGGAATCTCATCTATTTTAGGAGCAGTAAATTTTATTACAACTGTAATTAATATACGATCAGCTGGTATTACTCTTGATCGACTACCTTTATTTGTGTGATCAGTAGTAATTACAGCTATTTTATTACTTCTTTCTCTACCCGTATTAGCCGGAGCTATTACTATATTATTAACAGACCGAAATTTAAATACATCTTTTTTTGATCCAATTGGAGGGGGAGACCCTATTTTATATCAA

> Sample 3.1

TTGGATCTGGAATAGTCGGAACTTCACTAAGAGTTTTAATTCGTATTGAACTTAGACATCCTGGTATATTTATTGGAAATGATCAAATTTATAATGTAATTGTTACTGCTCATGCTTTTATTATAATTTTTTTTATAGTAATACCTATCATAATTGGAGGATTTGGAAACTGACTAGTACCCTTAATACTAGGAGCCCCTGATATAGCTTTTCCTCGAATAAATAATATAAGTTTTTGAATATTACCCCCCTCTTTAACACTGCTGCTTTCTAGTTCTATAGTAGAAAACGGAGCTGGAACAGGGTGAACGGTTTATCCTCCTCTTTCTTCTGGAACAGCTCATGCTGGGGCTTCAGTTGATTTAGCAATTTTTTCTTTACATTTAGCGGGAATCTCATCTATTTTAGGAGCAGTAAATTTTATTACAACTGTAATTAATATACGATCAGCTGGTATTACTCTTGATCGACTACCTTTATTTGTGTGATCAGTAGTAATTACAGCTATTTTATTACTTCTTTCTCTACCCGTATTAGCCGGAGCTATTACTATATTATTAACAGACCGAAATTTAAATACATCTTTTTTTGATCCAATTGGAGGGGGAGACCCTATTTTATATCAACATTT

> Sample 4.1

TCGGTATTTGATCTGGAATAGTCGGAACTTCACTAAGAGTTTTAATTCGTATTGAACTTAGACATCCTGGTATATTTATTGGAAATGATCAAATTTATAATGTAATTGTTACTGCTCATGCTTTTATTATAATTTTTTTTATAGTAATACCTATCATAATTGGAGGATTTGGAAACTGACTAGTACCCTTAATACTAGGAGCCCCTGATATAGCTTTTCCTCGAATAAATAATATAAGTTTTTGAATATTACCCCCCTCTTTAACACTGCTGCTTTCTAGTTCTATAGTAGAAAACGGAGCTGGAACAGGGTGAACGGTTTATCCTCCTCTTTCTTCTGGAACAGCTCATGCTGGGGCTTCAGTTGATTTAGCAATTTTTTCTTTACATTTAGCGGGAATCTCATCTATTTTAGGAGCAGTAAATTTTATTACAACTGTAATTAATATACGATCAGCTGGTATTACTCTTGATCGACTACCTTTATTTGTGTGATCAGTAGTAATTACAGCTATTTTATTACTTCTTTCTCTACCCGTATTAGCCGGAGCTATTACTATATTATTAACAGACCGAAATTTAAATACATCTTTTTTTGATCCAATTGGAGGGGGAGACCC

> Sample 5.1

TTTATTTTCGGTATTTGATCTGGAATAGTCGGAACTTCACTAAGAGTTTTAATTCGTATTGAACTTAGACATCCTGGTATATTTATTGGAAATGATCAAATTTATAATGTAATTGTTACTGCTCATGCTTTTATTATAATTTTTTTTATAGTAATACCTATCATAATTGGAGGATTTGGAAACTGACTAGTACCCTTAATACTAGGAGCCCCTGATATAGCTTTTCCTCGAATAAATAATATAAGTTTTTGAATATTACCCCCCTCTTTAACACTGCTGCTTTCTAGTTCTATAGTAGAAAACGGAGCTGGAACAGGGTGAACGGTTTATCCTCCTCTTTCTTCTGGAACAGCTCATGCTGGGGCTTCAGTTGATTTAGCAATTTTTTCTTTACATTTAGCGGGAATCTCATCTATTTTAGGAGCAGTAAATTTTATTACAACTGTAATTAATATACGATCAGCTGGTATTACTCTTGATCGACTACCTTTATTTGTGTGATCAGTAGTAATTACAGCTATTTTATTACTTCTTTCTCTACCCGTATTAGCCGGAGCTATTACTATATTATTAACAGACCGAAATTTAAATACATCTTTTTTTGATCCAATTGGAGGGGGAGACCCTATTTTATATCAACATTT

> Sample 6.1

ACTTTATTTTCGGTATTTGATCTGGAATAGTCGGAACTTCACTAAGAGTTTTAATTCGTATTGAACTTAGACATCCTGGTATATTTATTGGAAATGATCAAATTTATAATGTAATTGTTACTGCTCATGCTTTTATTATAATTTTTTTTATAGTAATACCTATCATAATTGGAGGATTTGGAAACTGACTAGTACCCTTAATACTAGGAGCCCCTGATATAGCTTTTCCTCGAATAAATAATATAAGTTTTTGAATATTACCCCCCTCTTTAACACTGCTGCTTTCTAGTTCTATAGTAGAAAACGGAGCTGGAACAGGGTGAACGGTTTATCCTCCTCTTTCTTCTGGAACAGCTCATGCTGGGGCTTCAGTTGATTTAGCAATTTTTTCTTTACATTTAGCGGGAATCTCATCTATTTTAGGAGCAGTAAATTTTATTACAACTGTAATTAATATACGATCAGCTGGTATTACTCTTGATCGACTACCTTTATTTGTGTGATCAGTAGTAATTACAGCTATTTTATTACTTCTTTCTCTACCCGTATTAGCCGGAGCTATTACTATATTATTAACAGACCGAAATTTAAATACATCTTTTTTTGATCCAATTGGAGGGGGAGACCCTATTTTA

> Sample 7.1

TTTGATCTGGAATAGTCGGAACTTCACTAAGAGTTTTAATTCGTATTGAACTTAGACATCCTGGTATATTTATTGGAAATGATCAAATTTATAATGTAATTGTTACTGCTCATGCTTTTATTATAATTTTTTTTATAGTAATACCTATCATAATTGGAGGATTTGGAAACTGACTAGTACCCTTAATACTAGGAGCCCCTGATATAGCTTTTCCTCGAATAAATAATATAAGTTTTTGAATATTACCCCCCTCTTTAACACTGCTGCTTTCTAGTTCTATAGTAGAAAACGGAGCTGGAACAGGGTGAACGGTTTATCCTCCTCTTTCTTCTGGAACAGCTCATGCTGGGGCTTCAGTTGATTTAGCAATTTTTTCTTTACATTTAGCGGGAATCTCATCTATTTTAGGAGCAGTAAATTTTATTACAACTGTAATTAATATACGATCAGCTGGTATTACTCTTGATCGACTACCTTTATTTGTGTGATCAGTAGTAATTACAGCTATTTTATTACTTCTTTCTCTACCCGTATTAGCCGGAGCTATTACTATATTATTAACAGACCGAAATTTAAATACATCTTTTTTTGATCCAATTGGAGGGGGAGACCC

> Sample 8.1

GGTATTTGATCTGGAATAGTCGGAACTTCACTAAGAGTTTTAATTCGTATTGAACTTAGACATCCTGGTATATTTATTGGAAATGATCAAATTTATAATGTAATTGTTACTGCTCATGCTTTTATTATAATTTTTTTTATAGTAATACCTATCATAATTGGAGGATTTGGAAACTGACTAGTACCCTTAATACTAGGAGCCCCTGATATAGCTTTTCCTCGAATAAATAATATAAGTTTTTGAATATTACCCCCCTCTTTAACACTGCTGCTTTCTAGTTCTATAGTAGAAAACGGAGCTGGAACAGGATGAACGGTTTATCCTCCTCTTTCTTCTGGAACAGCTCATGCTGGGGCTTCAGTTGATTTAGCAATTTTTTCTTTACATTTAGCGGGAATCTCATCTATTTTAGGAGCAGTAAATTTTATTACAACTGTAATTAATATACGATCAGCTGGTATTACTCTTGATCGACTACCTTTATTTGTGTGATCAGTAGTAATTACAGCTATTTTATTACTTCTTTCTCTACCCGTATTAGCCGGAGCTATTACTATATTATTAACAGACCGAAATTTAAATACATCTTTTTTTGATCCAATTGGAGGGGGAGACCC

> Sample 9.1

TTGATCTGGAATAGTCGGAACTTCACTAAGAGTTTTAATTCGTATTGAACTTAGACATCCTGGTATATTTATTGGAAATGATCAAATTTATAATGTAATTGTTACTGCTCATGCTTTTATTATAATTTTTTTTATAGTAATACCTATCATAATTGGAGGATTTGGAAACTGACTAGTACCCTTAATACTAGGAGCCCCTGATATAGCTTTTCCTCGAATAAATAATATAAGTTTTTGAATATTACCCCCCTCTTTAACACTGCTGCTTTCTAGTTCTATAGTAGAAAACGGAGCTGGAACAGGGTGAACGGTTTATCCTCCTCTTTCTTCTGGAACAGCTCATGCTGGGGCTTCAGTTGATTTAGCAATTTTTTCTTTACATTTAGCGGGAATCTCATCTATTTTAGGAGCAGTAAATTTTATTACAACTGTAATTAATATACGATCAGCTGGTATTACTCTTGATCGACTACCTTTATTTGTGTGATCAGTAGTAATTACAGCTATTTTATTACTTCTTTCTCTACCCGTATTAGCCGGAGCTATTACTATATTATTAACAGACCGAAATTTAAATACATCTTTTTTTGATCCAATTGGAGGGGGAGACCCTATTTAAT

> Sample 10.1

TTATTTTCGGTATTTGATCTGGAATAGTCGGAACTTCACTAAGAGTTTTAATTCGTATTGAACTTAGACATCCTGGTATATTTATTGGAAATGATCAAATTTATAATGTAATTGTTACTGCTCATGCTTTTATTATAATTTTTTTTATAGTAATACCTATCATAATTGGAGGATTTGGAAACTGACTAGTACCCTTAATACTAGGAGCCCCTGATATAGCTTTTCCTCGAATAAATAATATAAGTTTTTGAATATTACCCCCCTCTTTAACACTGCTGCTTTCTAGTTCTATAGTAGAAAACGGAGCTGGAACAGGGTGAACGGTTTATCCTCCTCTTTCTTCTGGAACAGCTCATGCTGGGGCTTCAGTTGATTTAGCAATTTTTTCTTTACATTTAGCGGGAATCTCATCTATTTTAGGAGCAGTAAATTTTATTACAACTGTAATTAATATACGATCAGCTGGTATTACTCTTGATCGACTACCTTTATTTGTGTGATCAGTAGTAATTACAGCTATTTTATTACTTCTTTCTCTACCCGTATTAGCCGGAGCTATTACTATATTATTAACAGACCGAAATTTAAATACATCTTTTTTTGATCCAATTGGAGGGGGAGGACCCC

> Sample 11.1

TTGATCTGGAATAGTCGGAACTTCACTAAGAGTTTTAATTCGTATTGAACTTAGACATCCTGGTATATTTATTGGAAATGATCAAATTTATAATGTAATTGTTACTGCTCATGCTTTTATTATAATTTTTTTTATAGTAATACCTATCATAATTGGAGGATTTGGAAACTGACTAGTACCCTTAATACTAGGAGCCCCTGATATAGCTTTTCCTCGAATAAATAATATAAGTTTTTGAATATTACCCCCCTCTTTAACACTGCTGCTTTCTAGTTCTATAGTAGAAAACGGAGCTGGAACAGGGTGAACGGTTTATCCTCCTCTTTCTTCTGGAACAGCTCATGCTGGGGCTTCAGTTGATTTAGCAATTTTTTCTTTACATTTAGCGGGAATCTCATCTATTTTAGGAGCAGTAAATTTTATTACAACTGTAATTAATATACGATCAGCTGGTATTACTCTTGATCGACTACCTTTATTTGTGTGATCAGTAGTAATTACAGCTATTTTATTACTTCTTTCTCTACCCGTATTAGCCGGAGCTATTACTATATTATTAACAGACCGAAATTTAAATACATCTTTTTTTGATCCAATTGGAGGGGGAGACC

> Sample 12.1

TTGATCCTGGAATAGTCGGAACTTCACTAAGAGTTTTAATTCGTATTGAACTTAGACATCCTGGTATATTTATTGGAAATGATCAAATTTATAATGTAATTGTTACTGCTCATGCTTTTATTATAATTTTTTTTATAGTAATACCTATCATAATTGGAGGATTTGGAAACTGACTAGTACCCTTAATACTAGGAGCCCCTGATATAGCTTTTCCTCGAATAAATAATATAAGTTTTTGAATATTACCCCCCTCTTTAACACTGCTGCTTTCTAGTTCTATAGTAGAAAACGGAGCTGGAACAGGATGAACGGTTTATCCTCCTCTTTCTTCTGGAACAGCTCATGCTGGGGCTTCAGTTGATTTAGCAATTTTTTCTTTACATTTAGCGGGAATCTCATCTATTTTAGGAGCAGTAAATTTTATTACAACTGTAATTAATATACGATCAGCTGGTATTACTCTTGATCGACTACCTTTATTTGTGTGATCAGTAGTAATTACAGCTATTTTATTACTTCTTTCTCTACCCGTATTAGCCGGAGCTATTACTATATTATTAACAGACCGAAATTTAAATAC

> Sample 13.1

TCAGGTATTTGATCTGGAATAGTCGGAACTTCACTAAGAGTTTTAATTCGTATTGAACTTAGACATCCTGGTATATTTATTGGAAATGATCAAATTTATAATGTAATTGTTACTGCTCATGCTTTTATTATAATTTTTTTTATAGTAATACCTATCATAATTGGAGGATTTGGAAACTGACTAGTACCCTTAATACTAGGAGCCCCTGATATAGCTTTTCCTCGAATAAATAATATAAGTTTTTGAATATTACCCCCCTCTTTAACACTGCTGCTTTCTAGTTCTATAGTAGAAAACGGAGCTGGAACAGGGTGAACGGTTTATCCTCCTCTTTCTTCTGGAACAGCTCATGCTGGGGCTTCAGTTGATTTAGCAATTTTTTCTTTACATTTAGCGGGAATCTCATCTATTTTAGGAGCAGTAAATTTTATTACAACTGTAATTAATATACGATCAGCTGGTATTACTCTTGATCGACTACCTTTATTTGTGTGATCAGTAGTAATTACAGCTATTTTATTACTTCTTTCTCTACCCGTATTAGCCGGAGCTATTACTATATTATTAACAGACCGAAATTTAAATACATCTTTTTTTGATCCAATTGGAGGGGGAGACCCTATTTTATATCAACATTTATTTTGATTTTTTGGTCACCCTGGAAGTTTAA

> Sample 14.1

ACATTCGGTATTTGATCTGGAATAGTCGGAACTTCACTAAGAGTTTTAATTCGTATTGAACTTAGACATCCTGGTATATTTATTGGAAATGATCAAATTTATAATGTAATTGTTACTGCTCATGCTTTTATTATAATTTTTTTTATAGTAATACCTATCATAATTGGAGGATTTGGAAACTGACTAGTACCCTTAATACTAGGAGCCCCTGATATAGCTTTTCCTCGAATAAATAATATAAGTTTTTGAATATTACCCCCCTCTTTAACACTGCTGCTTTCTAGTTCTATAGTAGAAAACGGAGCTGGAACAGGGTGAACGGTTTATCCTCCTCTTTCTTCTGGAACAGCTCATGCTGGGGCTTCAGTTGATTTAGCAATTTTTTCTTTACATTTAGCGGGAATCTCATCTATTTTAGGAGCAGTAAATTTTATTACAACTGTAATTAATATACGATCAGCTGGTATTACTCTTGATCGACTACCTTTATTTGTGTGATCAGTAGTAATTACAGCTATTTTATTACTTCTTTCTCTACCCGTATTAGCCGGAGCTATTACTATATTATTAACAGACCGAAATTTAAATACATCTTTTTTTGATCCAATTGGAGGGGGAGACCCTATTTTATATCAACATTTATTTTGATTTTTTGGTCACCCTGGAAGTTAAA

> Sample 15.1

TCGGTATTTGATCTGGATAGTCGGAACTTCACTAAGAGTTTTAATTCGTATTGAACTTAGACATCCTGGTATATTTATTGGAAATGATCAAATTTATAATGTAATTGTTACTGCTCATGCTTTTATTATAATTTTTTTTATAGTAATACCTATCATAATTGGAGGATTTGGAAACTGACTAGTACCCTTAATACTAGGAGCCCCTGATATAGCTTTTCCTCGAATAAATAATATAAGTTTTTGAATATTACCCCCCTCTTTAACACTGCTGCTTTCTAGTTCTATAGTAGAAAACGGAGCTGGAACAGGGTGAACGGTTTATCCTCCTCTTTCTTCTGGAACAGCTCATGCTGGGGCTTCAGTTGATTTAGCAATTTTTTCTTTACATTTAGCGGGAATCTCATCTATTTTAGGAGCAGTAAATTTTATTACAACTGTAATTAATATACGATCAGCTGGTATTACTCTTGATCGACTACCTTTATTTGTGTGATCAGTAGTAATTACAGCTATTTTATTACTTCTTTCTCTACCCGTATTAGCCGGAGCTATTACTATATTATTAACAGACCGAAATTTAAATACATCTTTTTTTGATCCAATTGGAGGGGGAGACCCTATTTTATATCAACATTTATTTTGATTTTTTGGTCACCCTGGGAAGTTAAA

> Sample 16.1

CATGCATTTGATCTGGAATAGTCGGAACTTCACTAAGAGTTTTAATTCGTATTGAACTTAGACATCCTGGTATATTTATTGGAAATGATCAAATTTATAATGTAATTGTTACTGCTCATGCTTTTATTATAATTTTTTTTATAGTAATACCTATCATAATTGGAGGATTTGGAAACTGACTAGTACCCTTAATACTAGGAGCCCCTGATATAGCTTTTCCTCGAATAAATAATATAAGTTTTTGAATATTACCCCCCTCTTTAACACTGCTGCTTTCTAGTTCTATAGTAGAAAACGGAGCTGGAACAGGGTGAACGGTTTATCCTCCTCTTTCTTCTGGAACAGCTCATGCTGGGGCTTCAGTTGATTTAGCAATTTTTTCTTTACATTTAGCGGGAATCTCATCTATTTTAGGAGCAGTAAATTTTATTACAACTGTAATTAATATACGATCAGCTGGTATTACTCTTGATCGACTACCTTTATTTGTGTGATCAGTAGTAATTACAGCTATTTTATTACTTCTTTCTCTACCCGTATTAGCCGGAGCTATTACTATATTATTAACAGACCGAAATTTAAATACATCTTTTTTTGATCCAATTGGAGGGGGAGACCCTATTTTATATCAACATTTATTTTGATTTTTTGGTCACCTGGGAAGTTAAAA

> Sample 17.1

CCGAGTATTTGATCTGGATAGTCGGAACTTCACTAAGAGTTTTAATTCGTATTGAACTTAGACATCCTGGTATATTTATTGGAAATGATCAAATTTATAATGTAATTGTTACTGCTCATGCTTTTATTATAATTTTTTTTATAGTAATACCTATCATAATTGGAGGATTTGGAAACTGACTAGTACCCTTAATACTAGGAGCCCCTGATATAGCTTTTCCTCGAATAAATAATATAAGTTTTTGAATATTACCCCCCTCTTTAACACTGCTGCTTTCTAGTTCTATAGTAGAAAACGGAGCTGGAACAGGGTGAACGGTTTATCCTCCTCTTTCTTCTGGAACAGCTCATGCTGGGGCTTCAGTTGATTTAGCAATTTTTTCTTTACATTTAGCGGGAATCTCATCTATTTTAGGAGCAGTAAATTTTATTACAACTGTAATTAATATACGATCAGCTGGTATTACTCTTGATCGACTACCTTTATTTGTGTGATCAGTAGTAATTACAGCTATTTTATTACTTCTTTCTCTACCCGTATTAGCCGGAGCTATTACTATATTATTAACAGACCGAAATTTAAATACATCTTTTTTTGATCCAATTGGAGGGGGAGACCCTATTTTATATCAACATTTATTTTGATTTTTTGGTCACCTGGAAGTTTAA

> Sample 18.1

GACGTCATTTTCGGTATTTGATCTGGAATAGTCGGAACTTCACTAAGAGTTTTAATTCGTATTGAACTTAGACATCCTGGTATATTTATTGGAAATGATCAAATTTATAATGTAATTGTTACTGCTCATGCTTTTATTATAATTTTTTTTATAGTAATACCTATCATAATTGGAGGATTTGGAAACTGACTAGTACCCTTAATACTAGGAGCCCCTGATATAGCTTTTCCTCGAATAAATAATATAAGTTTTTGAATATTACCCCCCTCTTTAACACTGCTGCTTTCTAGTTCTATAGTAGAAAACGGAGCTGGAACAGGGTGAACGGTTTATCCTCCTCTTTCTTCTGGAACAGCTCATGCTGGGGCTTCAGTTGATTTAGCAATTTTTTCTTTACATTTAGCGGGAATCTCATCTATTTTAGGAGCAGTAAATTTTATTACAACTGTAATTAATATACGATCAGCTGGTATTACTCTTGATCGACTACCTTTATTTGTATGATCAGTAGTAATTACAGCTATTTTATTACTTCTTTCTCTACCCGTATTAGCCGGAGCTATTACTATATTATTAACAGACCGAAATTTAAATACATCTTTTTTTGATCCAATTGGAGGGGGAGACCCTATTTTATATCAACATTTATTTTGATTTTTTGGTCACCCTGGAAGTTTAA

> Sample 19.1

CCTTTTCTTTTATTTTCGGTATTTGATCTGGAATAGTCGGAACTTCACTAAGAGTTTTAATTCGTATTGAACTTAGACATCCTGGTATATTTATTGGAAATGATCAAATTTATAATGTAATTGTTACTGCTCATGCTTTTATTATAATTTTTTTTATAGTAATACCTATCATAATTGGAGGATTTGGAAACTGACTAGTACCCTTAATACTAGGAGCCCCTGATATAGCTTTTCCTCGAATAAATAATATAAGTTTTTGAATATTACCCCCCTCTTTAACACTGCTGCTTTCTAGTTCTATAGTAGAAAACGGAGCTGGAACAGGGTGAACGGTTTATCCTCCTCTTTCTTCTGGAACAGCTCATGCTGGGGCTTCAGTTGATTTAGCAATTTTTTCTTTACATTTAGCGGGAATCTCATCTATTTTAGGAGCAGTAAATTTTATTACAACTGTAATTAATATACGATCAGCTGGTATTACTCTTGATCGACTACCTTTATTTGTGTGATCAGTAGTAATTACAGCTATTTTATTACTTCTTTCTCTACCCGTATTAGCCGGAGCTATTACTATATTATTAACAGACCGAAATTTAAATACATCTTTTTTTGATCCAATTGGAGGGGGAGACCCTATTTTATATCAACATTTATTTTGATTTTTTGGTCACCCTGGAAGTTTAA

> Sample 20.1

TCCGTTTACTTTCGGTATTTGATCTGGATAGTCGGAACTTCACTAAGAGTTTTAATTCGTATTGAACTTAGACATCCTGGTATATTTATTGGAAATGATCAAATTTATAATGTAATTGTTACTGCTCATGCTTTTATTATAATTTTTTTTATAGTAATACCTATCATAATTGGAGGATTTGGAAACTGACTAGTACCCTTAATACTAGGAGCCCCTGATATAGCTTTTCCTCGAATAAATAATATAAGTTTTTGAATATTACCCCCCTCTTTAACACTGCTGCTTTCTAGTTCTATAGTAGAAAACGGAGCTGGAACAGGGTGAACGGTTTATCCTCCTCTTTCTTCTGGAACAGCTCATGCTGGGGCTTCAGTTGATTTAGCAATTTTTTCTTTACATTTAGCGGGAATCTCATCTATTTTAGGAGCAGTAAATTTTATTACAACTGTAATTAATATACGATCAGCTGGTATTACTCTTGATCGACTACCTTTATTTGTGTGATCAGTAGTAATTACAGCTATTTTATTACTTCTTTCTCTACCCGTATTAGCCGGAGCTATTACTATATTATTAACAGACCGAAATTTAAATACATCTTTTTTTGATCCAATTGGAGGGGGAGACCCTATTTTATATCAACATTTATTTTGATTTTTTGGTCACCCTGGAAGTTAAA

> Sample 21.1

CAAAAACGTTTATTTTCGGTATTTGATCTGGATAGTCGGAACTTCACTAAGAGTTTTAATTCGTATTGAACTTAGACATCCTGGTATATTTATTGGAAATGATCAAATTTATAATGTAATTGTTACTGCTCATGCTTTTATTATAATTTTTTTTATAGTAATACCTATCATAATTGGAGGATTTGGAAACTGACTAGTACCCTTAATACTAGGAGCCCCTGATATAGCTTTTCCTCGAATAAATAATATAAGTTTTTGAATATTACCCCCCTCTTTAACACTGCTGCTTTCTAGTTCTATAGTAGAAAACGGAGCTGGAACAGGGTGAACGGTTTATCCTCCTCTTTCTTCTGGAACAGCTCATGCTGGGGCTTCAGTTGATTTAGCAATTTTTTCTTTACATTTAGCGGGAATCTCATCTATTTTAGGAGCAGTAAATTTTATTACAACTGTAATTAATATACGATCAGCTGGTATTACTCTTGATCGACTACCTTTATTTGTGTGATCAGTAGTAATTACAGCTATTTTATTACTTCTTTCTCTACCCGTATTAGCCGGAGCTATTACTATATTATTAACAGACCGAAATTTAAATACATCTTTTTTTGATCCAATTGGAGGGGGAGACCCTATTTTATATCAACATTTATTTTGATTTTTTGGTCACCCTGGAAGTTTAA

> Sample 22.1

TAAACTTTGATCTGGAATAGTCGGAACTTCACTAAGAGTTTTAATTCGTATTGAACTTAGACATCCTGGCATATTTATTGGAAATGATCAAATTTATAATGTAATTGTTACTGCTCATGCTTTTATTATAATTTTTTTTATAGTAATACCTATCATAATTGGAGGATTTGGAAACTGACTAGTACCCTTAATACTAGGAGCCCCTGATATAGCTTTTCCTCGAATAAATAATATAAGTTTTTGAATATTACCCCCCTCTTTAACACTGCTGCTTTCTAGTTCTATAGTAGAAAACGGAGCTGGAACAGGGTGAACGGTTTATCCTCCTCTTTCTTCTGGAACAGCTCATGCTGGGGCTTCAGTTGATTTAGCAATTTTTTCTTTACATTTAGCGGGAATCTCATCTATTTTAGGAGCAGTAAATTTTATTACAACTGTAATTAATATACGATCAGCTGGTATTACTCTTGATCGACTACCTTTATTTGTGTGATCAGTAGTAATTACAGCTATTTTATTACTTCTTTCTCTACCCGTATTAGCCGGAGCTATTACTATATTATTAACAGACCGAAATTTAAATACATCTTTTTTTGATCCAATTGGAGGGGGAGACCCTATTTTATATCAACATTTATTTTGATTTTTTGGTCACCCTGGGAAGTTATA

> Sample1.2

TCTGGTGCTTGAGCCGGATAGTAGGAACTTCTTTAAGTATTCTAATTCGAGCTGAATTAAGTCATCCCGGTGCTTTTATTGGAGATGATCAAATTTATAATGTTATTGTAACTGCTCATGCTTTTATTATAATTTTTTTTATAGTAATGCCTATTATAATTGGAGGATTCGGAAATTGATTAGTTCCTTTAATATTAGGAGCTCCTGATATAGCCTTTCCTCGAATAAATAATATAAGTTTTTGAATACTACCTCCTTCATTAACATTACTAATTTCAAGAAGTATAGTAGAAAATGGAGCTGGAACTGGGTGAACTGTTTATCCCCCTCTTTCATCTGGAATTGCTCATGCTGGAGCATCAGTAGATTTAGCTATTTTTTCTTTACATTTAGCTGGGATTTCATCAATTTTAGGAGCAGTAAATTTTATTACAACTGTAATTAATATACGATCTCCAGGAATTACATTAGATCGAATACCTTTATTTGTTTGATCAGTAGTAATTACAGCAGTTTTATTACTTCTATCTTTACCAGTATTAGCCGGAGCTATTACTATACTTTTAACAGATCGAAATTTAAATACCTCATTCTTTGACCCAACTGGAGGAGGAGACCCTATTTTATATCAACATTTATTCTGATTTTTTGGTCACCCTGAAGTTTAAA

> Sample2.2

CTGAGTATAGTAGGAACTTCTTTAAGTATTCTAATTCGAGCTGAATTAGGTCATCCTGGTGCTTTTATTGGAGATGATCAAATTTATAATGTTATTGTAACAGCACATGCTTTTATTATAATTTTTTTTATAGTTATACCTATTATAATTGGAGGATTCGGAAATTGATTAGTTCCATTAATATTAGGAGCCCCTGATATAGCTTTTCCTCGAATAAATAATATAAGTTTTTGAATATTACCTCCTTCATTAACATTACTAATTTCTAGAAGTATAGTAGAAAATGGAGCAGGAACAGGATGAACTGTTTATCCCCCACTTTCATCTGGAATTGCTCATGCTGGAGCATCAGTAGATTTAGCTATTTTTTCATTACATTTAGCTGGGATTTCATCAATTTTAGGAGCAGTAAATTTTATTACAACTGTAATTAATATACGATCTCCAGGAATTACATTAGATCGAATACCTCTATTTGTTTGATCAGTAGTAATTACAGCAGTATTATTACTTTTATCTTTACCAGTATTAGCCGGAGCTATTACTATACTTTTAACAGATCGAAATTTAAATACCTCATTCTTTGATCCAGCTGGAGGAGGAGATCCTATTTTATATCAACATTTATTCTGATTTTTTGGTCACCTTGGAAAGTTTAAAGTAAAGC

>sample 1.3

CTCGTGGGTTACAAAAACAAAATAAAGATATTGGAACTTTATATTTTATTTTTGGTGCTTGAGCTGGAATAGTGGGAACTTCTTTAAGTATTTTAATTCGAACAGAATTAAATCACCCTGGAGTATTTATTGGAAATGATCAAATTTATAATGTAATTGTAACAGCTCATGCTTTTATTATAATTTTTTTTATAGTTATACCAATTATAATTGGAGGATTTGGAAATTGATTAGTACCCCTTATACTTGGAGCTCCAGATATAGCCTTCCCTCGAATAAATAATATAAGTTTTTGAATATTACCCCCTTCATTAACTCTACTAATTTCAAGTTCTTTAGTAGAAACAGGAGCTGGAACTGGGTGAACCGTTTATCCTCCTTTATCTTCTGGAACTGCTCATGCTGGAGCTTCTGTTGATTTAGCTATTTTCTCTCTTCATTTAGCAGGTATTTCTTCTATTTTGGGAGCAGTAAATTTTATTACAACTGTAATTAATATACGATCATCAGGGATTACTCTCGATCGATTACCCTTATTTGTTTGATCTGTTGTTATTACAGCTATTTTACTTCTTCTTTCTTTACCAGTTTTAGCAGGAGCTATTACTATACTATTAACTGATCGGAATTTAAATACCTCATTCTTTGACCCAATCGGAGGAGGCAG

>sample 2.3

CGGGGGGTGCCTTTGAAGGCTGGAATAGTGGGAACTTCTTTAAGTATTTTAATTCGAACAGAATTAAATCACCCTGGAGTATTTATTGGAAATGATCAAATTTATAATGTAATTGTAACAGCTCATGCTTTTATTATAATTTTTTTTATAGTTATACCAATTATAATTGGAGGATTTGGAAATTGATTAGTACCCCTTATACTTGGAGCTCCAGATATAGCCTTCCCTCGAATAAATAATATAAGTTTTTGAATATTACCCCCTTCATTAACTCTACTAATTTCAAGTTCTTTAGTAGAAACAGGAGCTGGAACTGGGTGAACCGTTTATCCTCCTTTATCTTCTGGAACTGCTCATGCTGGAGCTTCTGTTGATTTAGCTATTTTCTCTCTTCATTTAGCAGGTATTTCTTCTATTTTGGGAGCAGTAAATTTTATTACAACTGTAATTAATATACGATCATCAGGGATTACTCTCGATCGATTACCCTTATTTGTTTGATCTGTTGTTATTACAGCTATTTTACTTCTTCTTTCTTTACCAGTTTTAGCAGGAGCTATTACTATACTATTAACTGATCGGAATTTAAATACCTCATTCTTTGACCCAATCGGAGGAGGAGACCCGATCTTATACCAACATTTATTTTGATTTTTTGGTCACCCTGAAGTTTAA

>sample 3.3

CGGGGCGGCTTGGAGGCTGGAATAGTGGGAACTTCTTTAAGTATTTTAATTCGAACAGAATTAAATCACCCTGGAGTATTTATTGGAAATGATCAAATTTATAATGTAATTGTAACAGCTCATGCTTTTATTATAATTTTTTTTATAGTTATACCAATTATAATTGGAGGATTTGGAAATTGATTAGTACCCCTTATACTTGGAGCTCCAGATATAGCCTTCCCTCGAATAAATAATATAAGTTTTTGAATATTACCCCCTTCATTAACTCTACTAATTTCAAGTTCTTTAGTAGAAACAGGAGCTGGAACTGGGTGAACCGTTTATCCTCCTTTATCTTCTGGAACTGCTCATGCTGGAGCTTCTGTTGATTTAGCTATTTTCTCTCTTCATTTAGCAGGTATTTCTTCTATTTTGGGAGCAGTAAATTTTATTACAACTGTAATTAATATACGATCATCAGGGATTACTCTCGATCGATTACCCTTATTTGTTTGATCTGTTGTTATTACAGCTATTTTACTTCTTCTTTCTTTACCAGTTTTAGCAGGAGCTATTACTATACTATTAACTGATCGGAATTTAAATACCTCATTCTTTGACCCAATCGGAGGAGGAGACCCGATCTTATACCAACATTTATTTTGATTTTTTGGTCACCCTGGAAGTTTAA

>sample 4.3

TTTTTTTTTTTGGGTGCCTTGAGCTGGAATAGTGGGAACTTCTTTAAGTATTTTAATTCGAACAGAATTAAATCACCCTGGAGTATTTATTGGAAATGATCAAATTTATAATGTAATTGTAACAGCTCATGCTTTTATTATAATTTTTTTTATAGTTATACCAATTATAATTGGAGGATTTGGAAATTGATTAGTACCCCTTATACTTGGAGCTCCAGATATAGCCTTCCCTCGAATAAATAATATAAGTTTTTGAATATTACCCCCTTCATTAACTCTACTAATTTCAAGTTCTTTAGTAGAAACAGGAGCTGGAACTGGGTGAACCGTTTATCCTCCTTTATCTTCTGGAACTGCTCATGCTGGAGCTTCTGTTGATTTAGCTATTTTCTCTCTTCATTTAGCAGGTATTTCTTCTATTTTGGGAGCAGTAAATTTTATTACAACTGTAATTAATATACGATCATCAGGGATTACTCTCGATCGATTACCCTTATTTGTTTGATCTGTTGTTATTACAGCTATTTTACTTCTTCTTTCTTTACCAGTTTTAGCAGGAGCTATTACTATACTATTAACTGATCGGAATTTAAATACCTCATTCTTTGACCCAATCGGAGGAGGAGACCCGATCTTATACCAACATTTATTTTGATTTTTTGGTCACCCTGAAGTTTAA

>sample 5.3

TAATTTTTTTTTTTTGGTGCTTGAGCTGGATAGTGGGAACTTCTTTAAGTATTTTAATTCGAACAGAATTAAATCACCCTGGAGTATTTATTGGAAATGATCAAATTTATAATGTAATTGTAACAGCTCATGCTTTTATTATAATTTTTTTTATAGTTATACCAATTATAATTGGAGGATTTGGAAATTGATTAGTACCCCTTATACTTGGAGCTCCAGATATAGCCTTCCCTCGAATAAATAATATAAGTTTTTGAATATTACCCCCTTCATTAACTCTACTAATTTCAAGTTCTTTAGTAGAAACAGGAGCTGGAACTGGGTGAACCGTTTATCCTCCTTTATCTTCTGGAACTGCTCATGCTGGAGCTTCTGTTGATTTAGCTATTTTCTCTCTTCATTTAGCAGGTATTTCTTCTATTTTGGGAGCAGTAAATTTTATTACAACTGTAATTAATATACGATCATCAGGGATTACTCTCGATCGATTACCCTTATTTGTTTGATCTGTTGTTATTACAGCTATTTTACTTCTTCTTTCTTTACCAGTTTTAGCAGGAGCTATTACTATACTATTAACTGATCGGAATTTAAATACCTCATTCTTTGACCCAATCGGAGGAGGAGACCCGATCTTATACCAACATTTATTTTGATTTTTTGGTCACCCTGGAAGTTTA

>sample 6.3

TATTTTTAATTTTTTTGGTGCTTGAGCTGGAATAGTGGGAACTTCTTTAAGTATTTTAATTCGAACAGAATTAAATCACCCTGGAGTATTTATTGGAAATGATCAAATTTATAATGTAATTGTAACAGCTCATGCTTTTATTATAATTTTTTTTATAGTTATACCAATTATAATTGGAGGATTTGGAAATTGATTAGTACCCCTTATACTTGGAGCTCCAGATATAGCCTTCCCTCGAATAAATAATATAAGTTTTTGAATATTACCCCCTTCATTAACTCTACTAATTTCAAGTTCTTTAGTAGAAACAGGAGCTGGAACTGGGTGAACCGTTTATCCTCCTTTATCTTCTGGAACTGCTCATGCTGGAGCTTCTGTTGATTTAGCTATTTTCTCTCTTCATTTAGCAGGTATTTCTTCTATTTTGGGAGCAGTAAATTTTATTACAACTGTAATTAATATACGATCATCAGGGATTACTCTCGATCGATTACCCTTATTTGTTTGATCTGTTGTTATTACAGCTATTTTACTTCTTCTTTCTTTACCAGTTTTAGCAGGAGCTATTACTATACTATTAACTGATCGGAATTTAAATACCTCATTCTTTGACCCAATCGGAGGAGGAGACCCGATCTTATACCAACATTTATTTTGATTTTTTGGTCACCCTGAAGTTTAA

>sample 7.3

TTTTTTGGGGTGCTTTGAGCTGGAATAGTGGGAACTTCTTTAAGTATTTTAATTCGAACAGAATTAAATCACCCTGGAGTATTTATTGGAAATGATCAAATTTATAATGTAATTGTAACAGCTCATGCTTTTATTATAATTTTTTTTATAGTTATACCAATTATAATTGGAGGATTTGGAAATTGATTAGTACCCCTTATACTTGGAGCTCCAGATATAGCCTTCCCTCGAATAAATAATATAAGTTTTTGAATATTACCCCCTTCATTAACTCTACTAATTTCAAGTTCTTTAGTAGAAACAGGAGCTGGAACTGGGTGAACCGTTTATCCTCCTTTATCTTCTGGAACTGCTCATGCTGGAGCTTCTGTTGATTTAGCTATTTTCTCTCTTCATTTAGCAGGTATTTCTTCTATTTTGGGAGCAGTAAATTTTATTACAACTGTAATTAATATACGATCATCAGGGATTACTCTCGATCGATTACCCTTATTTGTTTGATCTGTTGTTATTACAGCTATTTTACTTCTTCTTTCTTTACCAGTTTTAGCAGGAGCTATTACTATACTATTAACTGATCGGAATTTAAATACCTCATTCTTTGACCCAATCGGAGGAGGAGACCCGATCTTATACCAACATTTATTTTGATTTTTTGGTCACCCTGGAAGTTTAA

>sample 8.3

CGGGGGTGGGCCTTGAAGCCTGGAATAGTGGGAACTTCTTTAAGTATTTTAATTCGAACAGAATTAAATCACCCTGGAGTATTTATTGGAAATGATCAAATTTATAATGTAATTGTAACAGCTCATGCTTTTATTATAATTTTTTTTATAGTTATACCAATTATAATTGGAGGATTTGGAAATTGATTAGTACCCCTTATACTTGGAGCTCCAGATATAGCCTTCCCTCGAATAAATAATATAAGTTTTTGAATATTACCCCCTTCATTAACTCTACTAATTTCAAGTTCTTTAGTAGAAACAGGAGCTGGAACTGGGTGAACCGTTTATCCTCCTTTATCTTCTGGAACTGCTCATGCTGGAGCTTCTGTTGATTTAGCTATTTTCTCTCTTCATTTAGCAGGTATTTCTTCTATTTTGGGAGCAGTAAATTTTATTACAACTGTAATTAATATACGATCATCAGGGATTACTCTCGATCGATTACCCTTATTTGTTTGATCTGTTGTTATTACAGCTATTTTACTTCTTCTTTCTTTACCAGTTTTAGCAGGAGCTATTACTATACTATTAACTGATCGGAATTTAAATACCTCATTCTTTGACCCAATCGGAGGAGGAGACCCGATCTTATACCAACATTTATTTTGATTTTTTGGTCACCCTGGAAGTTTAA

>sample 9.3

CGGGGGTGCCTTTGGAGCCTGGAATAGTGGGAACTTCTTTAAGTATTTTAATTCGAACAGAATTAAATCACCCTGGAGTATTTATTGGAAATGATCAAATTTATAATGTAATTGTAACAGCTCATGCTTTTATTATAATTTTTTTTATAGTTATACCAATTATAATTGGAGGATTTGGAAATTGATTAGTACCCCTTATACTTGGAGCTCCAGATATAGCCTTCCCTCGAATAAATAATATAAGTTTTTGAATATTACCCCCTTCATTAACTCTACTAATTTCAAGTTCTTTAGTAGAAACAGGAGCTGGAACTGGGTGAACCGTTTATCCTCCTTTATCTTCTGGAACTGCTCATGCTGGAGCTTCTGTTGATTTAGCTATTTTCTCTCTTCATTTAGCAGGTATTTCTTCTATTTTGGGAGCAGTAAATTTTATTACAACTGTAATTAATATACGATCATCAGGGATTACTCTCGATCGATTACCCTTATTTGTTTGATCTGTTGTTATTACAGCTATTTTACTTCTTCTTTCTTTACCAGTTTTAGCAGGAGCTATTACTATACTATTAACTGATCGGAATTTAAATACCTCATTCTTTGACCCAATCGGAGGAGGAGACCCGATCTTATACCAACATTTATTTTGATTTTTTGGTCACCCTGGAAGTTTAA

>sample 10.3

GGGGTGGCTTTGGGAGCTGGAATAGTGGGAACTTCTTTAAGTATTTTAATTCGAACAGAATTAAATCACCCTGGAGTATTTATTGGAAATGATCAAATTTATAATGTAATTGTAACAGCTCATGCTTTTATTATAATTTTTTTTATAGTTATACCAATTATAATTGGAGGATTTGGAAATTGATTAGTACCCCTTATACTTGGAGCTCCAGATATAGCCTTCCCTCGAATAAATAATATAAGTTTTTGAATATTACCCCCTTCATTAACTCTACTAATTTCAAGTTCTTTAGTAGAAACAGGAGCTGGAACTGGGTGAACCGTTTATCCTCCTTTATCTTCTGGAACTGCTCATGCTGGAGCTTCTGTTGATTTAGCTATTTTCTCTCTTCATTTAGCAGGTATTTCTTCTATTTTGGGAGCAGTAAATTTTATTACAACTGTAATTAATATACGATCATCAGGGATTACTCTCGATCGATTACCCTTATTTGTTTGATCTGTTGTTATTACAGCTATTTTACTTCTTCTTTCTTTACCAGTTTTAGCAGGAGCTATTACTATACTATTAACTGATCGGAATTTAAATACCTCATTCTTTGACCCAATCGGAGGAGGAGACCCGATCTTATACCAACATTTATTTTGATTTTTTGGTCACCCTGAAGTTTA

>sample 11.3

TGGGGGGGGCCTTTTGAAGCCTGGGAAGTAAGTGGGAACTTCTTTAAGTATTTTAATTCGAACAGAATTAAATCACCCTGGAGTATTTATTGGAAATGATCAAATTTATAATGTAATTGTAACAGCTCATGCTTTTATTATAATTTTTTTTATAGTTATACCAATTATAATTGGAGGATTTGGAAATTGATTAGTACCCCTTATACTTGGAGCTCCAGATATAGCCTTCCCTCGAATAAATAATATAAGTTTTTGAATATTACCCCCTTCATTAACTCTACTAATTTCAAGTTCTTTAGTAGAAACAGGAGCTGGAACTGGGTGAACCGTTTATCCTCCTTTATCTTCTGGAACTGCTCATGCTGGAGCTTCTGTTGATTTAGCTATTTTCTCTCTTCATTTAGCAGGTATTTCTTCTATTTTGGGAGCAGTAAATTTTATTACAACTGTAATTAATATACGATCATCAGGGATTACTCTCGATCGATTACCCTTATTTGTTTGATCTGTTGTTATTACAGCTATTTTACTTCTTCTTTCTTTACCAGTTTTAGCAGGAGCTATTACTATACTATTAACTGATCGGAATTTAAATACCTCATTCTTTGACCCAATCGGAGGAGGAGACCCGATCTTATACCAACATTTATTTTGATTTTTTGGTCACCCTGGAAGTTTA

>sample 12.3

TGGGGTGGCTTTGAGGCCTGGAATAGTGGGAACTTCTTTAAGTATTTTAATTCGAACAGAATTAAATCACCCTGGAGTATTTATTGGAAATGATCAAATTTATAATGTAATTGTAACAGCTCATGCTTTTATTATAATTTTTTTTATAGTTATACCAATTATAATTGGAGGATTTGGAAATTGATTAGTACCCCTTATACTTGGAGCTCCAGATATAGCCTTCCCTCGAATAAATAATATAAGTTTTTGAATATTACCCCCTTCATTAACTCTACTAATTTCAAGTTCTTTAGTAGAAACAGGAGCTGGAACTGGGTGAACCGTTTATCCTCCTTTATCTTCTGGAACTGCTCATGCTGGAGCTTCTGTTGATTTAGCTATTTTCTCTCTTCATTTAGCAGGTATTTCTTCTATTTTGGGAGCAGTAAATTTTATTACAACTGTAATTAATATACGATCATCAGGGATTACTCTCGATCGATTACCCTTATTTGTTTGATCTGTTGTTATTACAGCTATTTTACTTCTTCTTTCTTTACCAGTTTTAGCAGGAGCTATTACTATACTATTAACTGATCGGAATTTAAATACCTCATTCTTTGACCCAATCGGAGGAGGAGACCCGATCTTATACCAACATTTATTTTGATTTTTTGGTCACCCTGAAGTTTAA

>sample 13.3

ATTTAATTTTTTTTTGGGGTGCCTTGAGCTGGAATAGTGGGAACTTCTTTAAGTATTTTAATTCGAACAGAATTAAATCACCCTGGAGTATTTATTGGAAATGATCAAATTTATAATGTAATTGTAACAGCTCATGCTTTTATTATAATTTTTTTTATAGTTATACCAATTATAATTGGAGGATTTGGAAATTGATTAGTACCCCTTATACTTGGAGCTCCAGATATAGCCTTCCCTCGAATAAATAATATAAGTTTTTGAATATTACCCCCTTCATTAACTCTACTAATTTCAAGTTCTTTAGTAGAAACAGGAGCTGGAACTGGGTGAACCGTTTATCCTCCTTTATCTTCTGGAACTGCTCATGCTGGAGCTTCTGTTGATTTAGCTATTTTCTCTCTTCATTTAGCAGGTATTTCTTCTATTTTGGGAGCAGTAAATTTTATTACAACTGTAATTAATATACGATCATCAGGGATTACTCTCGATCGATTACCCTTATTTGTTTGATCTGTTGTTATTACAGCTATTTTACTTCTTCTTTCTTTACCAGTTTTAGCAGGAGCTATTACTATACTATTAACTGATCGGAATTTAAATACCTCATTCTTTGACCCAATCGGAGGAGGAGACCCGATCTTATACCAACATTTATTTTGATTTTTTGGTCACCCTGAAAGTTTAA

>sample 14.3

ATTTTAATTTTTTTGGTGCTTGAGCTGGAATAGTGGGAACTTCTTTAAGTATTTTAATTCGAACAGAATTAAATCACCCTGGAGTATTTATTGGAAATGATCAAATTTATAATGTAATTGTAACAGCTCATGCTTTTATTATAATTTTTTTTATAGTTATACCAATTATAATTGGAGGATTTGGAAATTGATTAGTACCCCTTATACTTGGAGCTCCAGATATAGCCTTCCCTCGAATAAATAATATAAGTTTTTGAATATTACCCCCTTCATTAACTCTACTAATTTCAAGTTCTTTAGTAGAAACAGGAGCTGGAACTGGGTGAACCGTTTATCCTCCTTTATCTTCTGGAACTGCTCATGCTGGAGCTTCTGTTGATTTAGCTATTTTCTCTCTTCATTTAGCAGGTATTTCTTCTATTTTGGGAGCAGTAAATTTTATTACAACTGTAATTAATATACGATCATCAGGGATTACTCTCGATCGATTACCCTTATTTGTTTGATCTGTTGTTATTACAGCTATTTTACTTCTTCTTTCTTTACCAGTTTTAGCAGGAGCTATTACTATACTATTAACTGATCGGAATTTAAATACCTCATTCTTTGACCCAATCGGAGGAGGAGACCCGATCTTATACCAACATTTATTTTGATTTTTTGGTCACCCTGAAGTTTAA
